# Multi-scale, numerical modeling of spatio-temporal signaling in cone phototransduction

**DOI:** 10.1371/journal.pone.0219848

**Published:** 2019-07-25

**Authors:** Colin Klaus, Giovanni Caruso, Vsevolod V. Gurevich, Emmanuele DiBenedetto

**Affiliations:** 1 The Mathematical Biosciences Institute, The Ohio State University, Columbus, OH, United States of America; 2 ITC, Italian National Research Council, Rome, Italy; 3 Department of Pharmacology, Vanderbilt University, Nashville, TN, United States of America; 4 Department of Mathematics, Vanderbilt University, Nashville, TN, United States of America; Eye Hospital, Charité, GERMANY

## Abstract

Mammals have two types of photoreceptors, rods and cones. While rods are exceptionally sensitive and mediate vision at very low illumination levels, cones operate in daylight and are responsible for the bulk of visual perception in most diurnal animals, including humans. Yet the mechanisms of phototransduction in cones is understudied, largely due to unavailability of pure cone outer segment (COS) preparations. Here we present a novel mathematical model of cone phototransduction that explicitly takes into account complex cone geometry and its multiple physical scales, faithfully reproduces features of the cone response, and is orders of magnitude more efficient than the standard 3D diffusion model. This is accomplished through the mathematical techniques of homogenization and concentrated capacity. The homogenized model is then computationally implemented by finite element method. This homogenized model permits one to analyze the effects of COS geometry on visual transduction and lends itself to performing large numbers of numerical trials, as required for parameter analysis and the stochasticity of rod and cone signal transduction. Agreement between the nonhomogenized, (i.e., standard 3D), and homogenized diffusion models is reported along with their simulation times and memory costs. Virtual expression of rod biochemistry on cone morphology is also presented for understanding some of the characteristic differences between rods and cones. These simulations evidence that 3D cone morphology and ion channel localization contribute to biphasic flash response, i.e undershoot. The 3D nonhomogenized and homogenized models are contrasted with more traditional and coarser well-stirred and 1D longitudinal diffusion models. The latter are single-scale and do not explicitly account for the multi-scale geometry of the COS, unlike the 3D homogenized model. We show that simpler models exaggerate the magnitude of the current suppression, yield accelerated time to peak, and do not predict the local concentration of cGMP at the ionic channels.

## Introduction

Phototransduction is the signaling process used by rod and cone photoreceptor cells to convert light into an electrical response [1–9]. Although cones mediate most of visual perception in humans and diurnal animals, their study is hampered by unavailability of purified COS preparations from most sources and consequent lack of information on the concentration and activity of most signaling molecules in mammalian cones [2, 3]. Here we propose a mathematical model of cone phototransduction that partially bridges this gap and reveals salient features of cones that determine their function.

Rods and cones, while geometrically different, both exhibit a layered array of parallel, functionally independent disc-like folded lipidic plasma membranes called discs. Discs house the light receptor rhodopsin (Rh), the transducer G protein (G), and the effector phosphodiesterase (PDE), [10]. These diffuse but remain on the faces of discs, and the activation of photopigment by photons occurs on these discs. A COS can be modeled by a right circular truncated cone from which a portion of lateral surface has been removed. The remaining portion is the *closed margin* [11] or *closing sliver*. The plasma membrane infolds repeatedly (≈ 500 in striped bass, [12]), while remaining connected by the closed sliver, to form equally spaced, equally thin (≈ 10 − 15nm), parallel double layers [13]. The closed margin contains cyclic nucleotide (CNG)-gated channels. In the absence of light these are kept open by cGMP, allowing an influx of Na^+^ and Ca^2+^ ions. This influx is balanced by an exchanger that removes Ca^2+^, thereby generating a steady-state dark current J_dark_ (for salamander J_dark_ ≈ 50pA [1]). The *second messengers* cGMP (cyclic-guanosine monophosphate), and calcium (Ca^2+^) diffuse within the cytosol.

Light activated rhodopsin R* activates G-protein transducin, converting it into T*, which in turn activates PDE by binding, generating the active E*. This cascade is 2-dimensional, as it takes place *only* on the *activated layer/disc*. Diffusion of the second messengers, cGMP and Ca^2+^, is 3-dimensional as it takes place in the cytosol. Active E* hydrolyzes cGMP, thereby lowering its concentration. As cGMP migrates from the (CNG)-gated channels on the closing sliver, the channels close which lowers the current across the closing margin. Calcium reduction, due to the exchanger, increases cGMP production by stimulation of Ca^2+^-inhibited guanylyl cyclase (GC) and thus leads to reopening of the channels. Recovery requires phosphorylation of activated visual pigments (R*) by a kinase followed by arrestin (Arr) binding [14].

This cascade, well known for rods [8, 10, 15–24], is the same for cones, except that the various biochemical players (Rh, G-protein, PDE, etc) are replaced by their cone-specific counterparts with different biochemical properties [1, 2, 25].

Rods and cones are functionally different [2, 3, 9, 26]. Unlike rods, cones keep CNG channels open in continued illumination that bleaches their pigments, thereby providing the basis of continued daytime vision. Rods are maximally sensitive to light at the wavelength λ ≈ 500 nm, whereas cones express their own specific visual pigments. In humans blue cones show maximum sensitivity at λ_*B*_ ≈ 430 nm, red cones at λ_*R*_ ≈ 560 nm, green cones at λ_*G*_ ≈ 530 nm [27].

On the electrophysiological side, peak response of cones occurs much faster than in rods, their single photon response is much smaller, and their recovery is quicker [2, 28–30]. In mouse cones t_peak_ ≈ 70 ms and peak-response ≈ 20% of J_dark_, [26], whereas in mouse rods t_peak_ ≈ 185 ms and peak-response ≈ 5.3% of J_dark_ [20, 31].

Considerably less information, especially biochemical, is available for cones than for rods. This is partly due to their fragility and the difficulty in purifying a population of single cone type for biochemical study (in mice cones are only 3% of photoreceptors [26, 32]). This increases the value of investigating cone functions by a mathematical model, particularly with 3D space-resolution to reflect the intricate layered geometry.

The geometry of the COS exhibits at least two geometrical scales: the size of the COS (≈ 10 *μ*m), and width of the discs and the closing margin (≈ 10nm). The second messengers exist in the COS whose scale is ≈ 10 *μ*m, and diffuse in the disc-layers, and in closing sliver whose scale is ≈ 10nm.

We bridge across scales by the following process: First we model the various cone functions pointwise by expressing the known biochemical and biophysical processes on their own scale, in their own location (discs, cytoplasm, outer shell), and irrespective of the geometry of the COS. Volume-to-surface interactions are incorporated as needed (for example, hydrolysis of cGMP, which diffuses in the cytosol by surface bound [PDE*]). This yields a system of non-linearly coupled diffusion partial differential equations for the second messengers cGMP and Ca^2+^ in the layered geometry of the cytoplasm with properly balanced fluxes on the boundary of the layers and the outer membrane. Such a model defined on the native cone geometry is presented in Eqs 1–11 of **Methods**.

Then one mathematically takes the homogenized limit of this system [33–39]. In practice, the number of discs is mathematically increased to infinity, while progressively shrinking their thickness, in such a way that the ratio of the cytosolic volume versus the geometrical volume of the cone remains constant. The limit is a system of diffusion processes, each acting on its own “limiting domain” (volume, surface), where, however, the geometry of the cone has been simplified by theoretically removing the layered discs.

In rods this process has been successfully carried out in [34, 40–45]. Cones, however, require their own separate treatment because of their conical taper and because, in contrast to rods, the cytosol layers between membrane folds are not connected over their entire lateral side [11].

This approach has theoretical and computational advantages. Theoretically it precisely states the law of diffusion in thin, perpendicular domains while simplifying the geometry (Eqs 13–15). Computationally it reduces numerical errors and significantly reduces the run time. Simulations with the homogenized model run in ≈ 2 minutes on a desktop while simulations with the nonhomogenized 2-scale model in its native geometry run in about 20 hours at the Ohio Supercomputer Center (OSC).

To the best of our knowledge, existing mathematical models for cone phototransduction are well-stirred or transversally well-stirred, e.g. the cone is assimilated to a segment with all its functions lumped along its vertical axis [12]. These models cannot account for the full effect of cone geometry. To emphasize the relevance of the cone geometry to its functions, the two space-resolved models are contrasted with spatially well-stirred (GWS) and also 1D longitudinal (LWS) models. The GWS model assumes concentrations are uniform across the COS and are governed by global, biochemical mass balance. The LWS model simplifies the cone to a line along its vertical axis. Horizontal diffusion is disregarded. The trials show that models neglecting the full 3D geometry distort 2nd messenger profiles in space, overestimate the peak relative current drop by an average error factor of 3.59 compared to the homogenized model, and shift the time to peak ≈ 10*ms* earlier. It emerges that COS morphology and spatial localization of ion channels appear to promote biphasic flash response, with characteristic undershoot, in the simulated ten photon drop response while they did not in the mouse ROS. Further, the spatially reduced GWS and LWS models were found to alter undershoot-dynamics. Biphasic flash response has been observed in several species, for example [46, 47], although recently its prevalence has been questioned [48].

The main goal of this paper is to introduce such a homogenized model (HOM) as a limit of the space resolved nonhomogenized two-scale model (NHOM) and show that these two models essentially make the same predictions. Though cones typically function under high intensity light, we compare the models in cases of ten or fewer photon isomerizations. This is done because these are the hardest benchmarks for the models to reproduce each other, as these cases are the most spatially localized. Parameter analysis is not intended here. Though parameters are taken from the literature where possible, which often requires having to select parameter values across different species, they are only used to demonstrate the extent of agreement between model types. However, once this validation has been shown, the homogenized model becomes a computational tool to perform almost real time, virtual COS experiments, including parameter estimation and analysis as well as hypotheses testing.

## Materials and methods

### A 3D nonhomogenized model for the visual transduction cascade

#### Geometry

**Fig 1:**
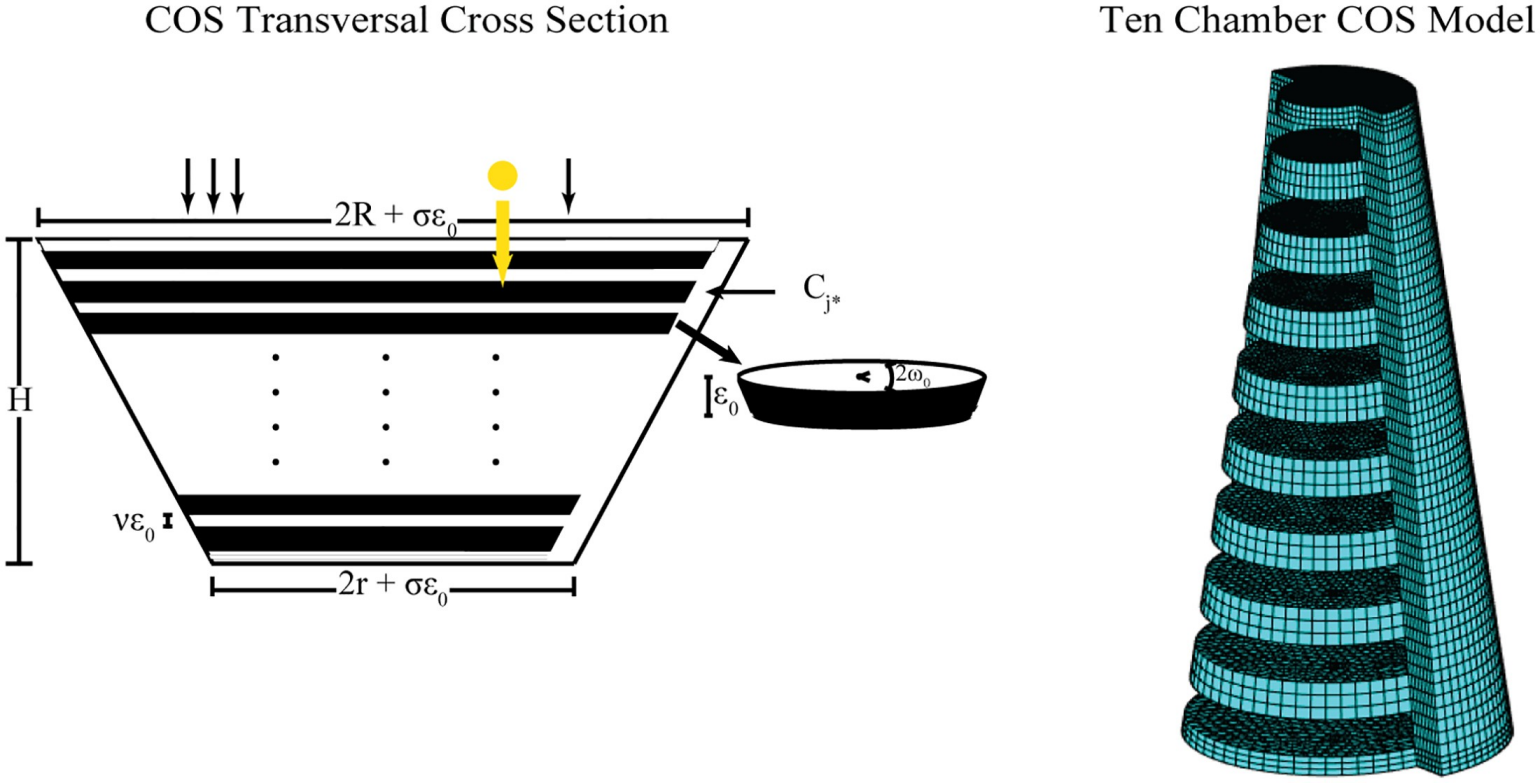
Cone outer segment geometry. On the left a transversal cross section of the COS is shown. The white space is the cytosol available to 2nd messenger diffusion. The black space is the lipidic discs whose surfaces carry the G-protein transduction biochemistry. On the right a low chamber finite element mesh produced by the NHOM matlab code is shown. Actual simulations were conducted with 500 chambers, but for illustrative purposes the ten chamber mesh is depicted. In this rendering, the space available to diffusion, the interdiscal layers, is shown in teal with a grid pattern. The sliver is also shown, and it is the only domain connecting adjacent layers. In mammalian cones, the sliver only extends over half of the circumference of the discs.

The COS in vertebrate photoreceptors exhibits a taper and stacked interdiscal layers of cytosol, which are connected through a margin closed to extracellular space that only partially extends over these layers’ rim. The closing sliver $S_{\epsilon_{o}}$ is a subset of the lateral boundary of Ω; it extends over *ω*_*o*_ radians of Ω’s horizontal circumference and has radial thickness *σϵ*_*o*_ for a parameter *σ* ∈ (0, 1) and a length *ϵ*_*o*_ of the order of 10*nm*. The COS is geometrically modeled by modifying a right circular cone Ω of radii 0 < *r* < *R* and height *H*. The interdiscal layers *I*_*j*_, for *j* = 1, …, *n*, and the closing sliver $S_{\epsilon_{o}}$ are what remains of Ω when the extracellular spaces *C*_*j*_ for *j* = 1, …, *n* − 1, between membrane folds within Ω are removed. Each *I*_*j*_ has thickness *νϵ*_*o*_, for some *ν* ∈ (0, 1), and it can be regarded as a horizontal slice of Ω. As such it is a truncated right circular cone of height *νϵ*_*o*_. The interdiscal layers *I*_*j*_ and their connecting closing sliver $S_{\epsilon_{o}}$ contain cytosol and the biochemical components of the visual transduction cascade. Their union is depicted by the white area in the cartoon of Fig 1.

The space available for diffusion of the second messengers cGMP and Ca^2+^ is

$$\begin{array}{c} {\tilde{\Omega }}_{\epsilon_{o}}={⋃}_{j=1}^{n}I_{j}\cup S_{\epsilon_{o}}=(\Omega -{⋃}_{j=1}^{n-1}C_{j})⋃S_{\epsilon_{o}}. \end{array}$$ (1)

From this construction one computes (S1 Appendix) that the proportion of volume of ${\tilde{\Omega }}_{\epsilon_{o}}$, available to 2nd messenger diffusion, to the volume of Ω, is independent of *ϵ*_*o*_ up to high order terms, i.e.,

$$\begin{array}{c} \frac{\text{vol}({\tilde{\Omega }}_{\epsilon_{o}})}{\text{vol}(\Omega )}=\frac{\nu }{1+\nu }+O\left(\epsilon_{o}^{2}\right). \end{array}$$ (2)

#### The nonhomogenized diffusion model (NHOM)

The second messengers cGMP and Ca^2+^ diffuse in the volumic cytosol ${\tilde{\Omega }}_{\epsilon_{o}}$. As they enter and exit the domain only through the signal transduction machinery and ion channels, which all reside at the cone membrane $\partial {\tilde{\Omega }}_{\epsilon_{o}}$, their concentrations satisfy a Fick’s diffusion law with respective diffusivities D_cG_ and D_Ca^2+^_ (in *μ*m^2^/s):

$$\begin{array}{c} \partial_{t}\text{[cG]}-\nabla \cdot \left({\text{D}}_{\text{cG}}\nabla \text{[cG]}\right)=0\;\;\text{and}\;\;\partial_{t}\text{[Ca}{}^{2+}\text{]}-\nabla \cdot \left({\text{D}}_{{\text{Ca}}^{2+}}\nabla \text{[Ca}{}^{2+}\text{]}\right)=0\;\;\text{in}\;\;{\tilde{\Omega }}_{\epsilon_{o}}. \end{array}$$ (3)

Here [cG] and [Ca^2+^] are volumic concentrations (in *μ*M). Activation is assumed to start from a basal, dark adapted state, so that [cG]_|*t* = 0_ = [cG]_dark_, and [Ca^2+^]_|*t*=0_ = [Ca^2+^]_dark_, where [cG]_dark_ and [Ca^2+^]_dark_ are the constant values of the dark adapted concentrations of cGMP and Ca^2+^ respectively.

The transduction biochemistry, which modulates cGMP concentration, resides on the cell membrane and is accordingly modeled with boundary flux terms. Even in the dark adapted state, there is hydrolysis and turnover of cGMP at all boundaries which contain PDE. Let [E] be the surface density of the effector PDE (in number of molecules per *μ*m^2^) on discal faces ∂*C*_*j*_. In accordance with section 2.2.1 of [43], the rate of *dark* cGMP hydrolysis per unit surface area is given by mass action with the *surface* rate constant k_*σ*;hyd_ (in *μ*m^3^/s):

$$\begin{array}{c} -{\text{k}}_{\sigma ;\text{hyd}}\left[\text{E}\right]\text{[cG]}=-\eta \beta_{\text{dark}}\text{[cG]}\;\;\text{on}\;\;\partial C_{j},\;\;\text{where}\;\;\eta =\frac{1}{2}\nu \epsilon_{o}. \end{array}$$ (4)

This dark hydrolysis is balanced by ongoing resynthesis of cGMP by guanylyl cyclase (GC), also on ∂*C*_*j*_. In turn, GC is stimulated by guanylate cyclase activating proteins (GCAPS) which are inhibited by Ca^2+^ [49–54], and so GC activity is Ca^2+^ dependent. The synthesis rate of cGMP follows a spatially localized Hill-type law owing to the binding of Ca^2+^ to GCAPS:

$$\begin{array}{c} \alpha \left(\text{[Ca}{}^{2+}\text{]}\right)=\alpha_{\text{min}}+\frac{\alpha_{\text{max}}-\alpha_{\text{min}}}{1+{\left(\text{[Ca}{}^{2+}\text{]}/{\text{K}}_{\text{cyc}}\right)}^{{\text{m}}_{\text{cyc}}}}\;\;\text{on}\;\partial C_{j}. \end{array}$$ (5)

The quantities *α*_min_ and *α*_max_ (in *μ*M/s) are respectively the least and greatest rate of cGMP synthesis by GC, m_cyc_ is the Hill coefficient, and K_cyc_ is the concentration for the half-maximal rate. Upon light activation, membrane bound PDE eventually switches to activated form, which is responsible for the hydrolysis of cGMP. PDE diffuses only on the membrane at ∂*C*_*j*_, unlike cGMP which diffuses in the cytosol. At the membrane face of photon capture, called $F_{i}^{*}$, there is the additional flux term, due to activation,

$$\begin{array}{c} -{\text{k}}_{\sigma ;\text{hyd}}^{*}\left[{\text{E}}^{*}\right]\text{[cG]}\;\;\text{on}\;F_{i}^{*}. \end{array}$$ (6)

Here ${\text{k}}_{\sigma ;\text{hyd}}^{*}$ (in *μ*m^3^/s) is the surface rate of hydroylysis by light activated phosphodiesterase, and [E*] (in number of molecules)/*μ*m^2^) is the surface concentration of of E*. Aggregating these flux terms and denoting by *δ*_*ij*_ the Kronecker delta to account for the site of activation, the boundary data for cGMP is given by

$$\begin{array}{c} {\text{D}}_{\text{cG}}\nabla \text{[cG]}\cdot \vec{n}=\{\begin{array}{cc} \eta \alpha \left(\text{[Ca}{}^{2+}\text{]}\right)-\eta \beta_{\text{dark}}\text{[cG]}-\delta_{ij}{\text{k}}_{\sigma ;\text{hyd}}^{*}\left[{\text{E}}^{*}\right]\text{[cG]} & \;\;\text{on}\;\;\partial C_{j}\\ 0 & \;\;\text{all}\;\text{other}\;\text{surfaces} \end{array} \end{array}$$ (7)

where $\vec{n}$ denotes the unit normal to the indicated surfaces, exterior to ${\tilde{\Omega }}_{\epsilon_{o}}$. Calcium enters the photoreceptor through cGMP-gated ion channels and exits the photoreceptor through electrogenic exchangers. Unless otherwise stated, both the channels and exchanger have been modeled as residing at the boundary $\partial S_{\epsilon_{o}}^{+}$ of the sliver $S_{\epsilon_{o}}$, exterior to Ω, and nowhere else. The calcium flux expresses these processes and, in accordance with [31], is given by

$$\begin{array}{c} {\text{D}}_{{\text{Ca}}^{2+}}\nabla \text{[Ca}{}^{2+}\text{]}\cdot \vec{n}=\{\begin{array}{cc} \frac{1}{{\text{B}}_{{\text{Ca}}^{2+}}F}(\frac{1}{2}f_{{\text{Ca}}^{2+}}{\text{J}}_{\text{cG}}\left(\text{[cG]}\right)-{\text{J}}_{\text{ex}}\left(\text{[Ca}{}^{2+}\text{]}\right)) & \;\;\text{on}\;\partial S_{\epsilon_{o}}^{+}\\ 0 & \;\;\text{on}\;\text{all}\;\text{other}\;\text{surfaces.} \end{array} \end{array}$$ (8)

Here B_Ca^2+^_ is the buffering power of the cytoplasm for calcium, F is Faraday’s constant and *f*_Ca^2+^_ is the fraction of current carried by calcium, which is known to be larger in cones than rods [55]. The terms J_ex_ and J_cG_ (in pA/*μ*m^2^) are the *current densities* of the exchanger and are due to the ionic cGMP-gated channels, relative to the surface $S_{\epsilon_{o}}^{+}$ where such current is produced. Their functional form, given by local Michaelis-Menten and Hill Laws [1], is

$$\begin{array}{c} {\text{J}}_{\text{ex}}\left(\text{[Ca}{}^{2+}\text{]}\right)=\frac{{\text{J}}_{\text{ex}}^{\text{sat}}}{\Sigma_{\text{cone}}}\frac{\text{[Ca}{}^{2+}\text{]}}{{\text{K}}_{\text{ex}}+\text{[Ca}{}^{2+}\text{]}},\;\;\text{and}\;\;{\text{J}}_{\text{cG}}\left(\text{[cG]}\right)=\frac{{\text{J}}_{\text{cG}}^{\text{max}}}{\Sigma_{\text{cone}}}\frac{{\text{[cG]}}^{{\text{m}}_{\text{cG}}}}{{\text{K}}_{\text{cG}}^{{\text{m}}_{\text{cG}}}+{\text{[cG]}}^{{\text{m}}_{\text{cG}}}}. \end{array}$$ (9)

The current values ${\text{J}}_{\text{ex}}^{\text{sat}}$ and ${\text{J}}_{\text{cG}}^{\text{max}}$ (in pA) are the maximum currents measured across the whole COS, respectively for either the exchanger as [Ca^2+^] becomes saturating or the cGMP-gated current as [cG] becomes saturating. The term *Σ*_cone_ is the area of $S_{\epsilon_{o}}^{+}$. This normalization assumes that the channels are distributed uniformly there. K_ex_ and K_cG_ are the concentrations for half-maximal current response in their respective equations. Some authors ([5, 56] and references therein) report that, unlike in rods, in cones the parameter K_cG_ varies sigmoidally with changes in intracellular calcium, and such a dependence is asserted to be physiologically significant. For the purposes of demonstrating numerical convergence, we have chosen to keep the mechanism of Eq 9 in the simulations shown here.

#### The activation mechanism

The processes of opsin activation by light, G-protein activation by opsin, and effector activation by G-protein generate the [E*] term of Eq 7. Denote by (*x*, *y*, *z*) the coordinates on ${\tilde{\Omega }}_{\epsilon_{o}}$, with the *z*-axis directed along the axis of the right, circular truncated cone Ω. It is assumed that cone opsin is activated by a photon at a fold located at some level *z*_*o*_ ∈ (0, *H*). Activated opsin, upon encounter on its random path *t* → **x**(*t*) with transducer G-protein, denoted by T, generates activated G-protein, T* which in turn diffuses throughout the activation disc and generates activated effector E* by mass action. To underscore that these processes do occur only on the 2-dimensional activated disc, denote by $\bar{x}=\left(x,y\right)$ the horizontal space variable on such a disc. Then in terms of $\bar{x}$, the governing equations are

$$\begin{array}{c} \begin{array}{cc} {}[{\text{T}}^{*}{]}_{t}-\nabla_{\bar{x}}\cdot \left({\text{D}}_{{\text{T}}^{*}}\nabla_{\bar{x}}\left[{\text{T}}^{*}\right]\right) & =\overset{N}{\underset{j=1}{\sum }}\nu_{j}\chi_{\left[t_{j-1},t_{j}\right]}\delta_{\text{x(t)}}-{\text{k}}_{\text{T}{\text{E}}^{*}}\left[\text{E}\right]\left[{\text{T}}^{*}\right]\\ {}[{\text{E}}^{*}{]}_{t}-\nabla_{\bar{x}}\cdot \left({\text{D}}_{{\text{E}}^{*}}\nabla_{\bar{x}}\left[{\text{E}}^{*}\right]\right) & ={\text{k}}_{\text{T}{\text{E}}^{*}}\left[\text{E}\right]\left[{\text{T}}^{*}\right]-{\text{k}}_{{\text{E}}^{*}}\left[{\text{E}}^{*}\right]. \end{array} \end{array}$$ (10)

Here $\nabla_{\bar{x}}$ denotes the gradient effected only with respect to the horizontal variables $\bar{x}$. The concentrations are surface densities (in number of molecules/*μ*m^2^), k_TE*_ is the rate of formation (in *μ*m^2^/s) of E* upon encounter with T*, and k_E*_ is the rate of depletion of E*. The *ν*_*j*_’s are the catalytic activity of (*j* − 1)-times phosphorylated opsin, and the [*t*_*j*−1_, *t*_*j*_] denote the random sojourn time intervals of the activated rhodopsin in each phosphorylation state. In keeping with [57], the catalytic activities *ν*_*j*_ decrease exponentially with the number *j* of phosphorylations, i.e.,

$$\begin{array}{c} \nu_{j}=\nu_{\text{R}{}^{*}{\text{T}}^{*}}e^{-k_{v}\left(j-1\right)} \end{array}$$ (11)

where *ν*_R*T*_ is the rate of formation of T* by activated non-phosphoryated opsin R* (j = 1), and *k*_*v*_ is a positive parameter. The random sojourn intervals [*t*_*j*−1_, *t*_*j*_] are distributed by a continuous time Markov chain described in [57]. Activated opsin R* is shut off by arrestin binding after a random number of phosphorylations. If it has (*j* − 1) phosphorylations, either it acquires another phosphate with probability λ_*j*_ or is bound by arrestin with probabiity *μ*_*j*_ and terminates. Transducer G-protein and effector PDE, in either their basal or activated state, do not exit the cone so that the fluxes of [T*] and [E*] across $\partial {\tilde{\Omega }}_{\epsilon_{o}}$ are zero.

The system Eq 10 describes activation due to a single isomerization on a fold at *z* = *z*_*o*_ level. Multiple simultaneous isomerizations on the same folds are described similarly where the term *δ*_**x**(*t*)_ is replaced by $\sum_{k=1}^{m}\delta_{x_{k}\left(t\right)}$, where *t* → **x**_*k*_(*t*) is the random path of the *k*-th activated opsin. Finally, multiple isomerizations on different discs located at levels *z* = *z*_*ℓ*_ for *ℓ* = 1, … *h* are modeled by an array of systems as in Eq 10, each written in the corresponding activated fold.

### The homogenized diffusion model (HOM)

The cone photoreceptor contains hundreds of finely spaced membrane folds [11]. Computer renderings of such a geometry require highly refined spatial discretizations to locate the folds. For example, the reported 2nd messenger NHOM simulations used a 500 chamber mesh with near seven million degrees of freedom. Following [41], the theoretical techniques of *homogenization and concentrated capacity* are administered to Eq 3 to yield a new model that removes the geometric complexity of ${\tilde{\Omega }}_{\epsilon_{o}}$. This new model still predicts COS diffusion but is defined on the simplified right circular truncated cone Ω with no layers.

Technically one replaces the physical parameter *ϵ*_*o*_ with an artificial parameter *ϵ* ∈ (0, *ϵ*_*o*_] and lets *ϵ* → 0, while “remembering” its original geometrical value. This is achieved by artificially increasing the number of folds and at the same time decreasing their thickness from *νϵ*_*o*_ to *νϵ* in such a way that the space available to diffusion is preserved up to a term of the order of *O*(*ϵ*^2^), in accordance with Eq 2. While all *C*_*j*_ and *I*_*j*_ become thinner to preserve this volume, the activation site at the face $F_{j}^{*}$ is kept at the same *z*-level [*z* = *z*_*o*_]. In the limit, the geometry is restored to the full Ω domain, no longer missing the *C*_*j*_ excisions. Simultaneously, the thin closing sliver $S_{\epsilon_{o}}$ retracts to *S*_*ϵ*_ and then, as *ϵ* → 0, to a 2-dimensional sliver *S* on the cone boundary, represented in cylindrical coordinates as

$$\begin{array}{c} S=\left\{\lambda \left(z\right),\theta ,z\right)|\theta \in \left[0,\omega_{o}\right],z\in \left[0,H\right]\},\;\;\text{with}\;\;\lambda \left(z\right)=r+\frac{R-r}{H}\;z. \end{array}$$ (12)

Preserving the limiting sliver *S*’s capacity for diffusion is mathematically realized by imposing, within the approximating slivers *S*_*ϵ*_, diffusion coefficients of the form D_cG_/*ϵ* and D_Ca^2+^_/*ϵ*. This is the contribution of the concentrated capacity technique. A similar rescaling is effected on the activated fold as its thickness *νϵ* → 0. This yields a family of systems of the form Eqs 1–9, with *ϵ*_*o*_ replaced by *ϵ*. As *ϵ* → 0 the mathematical homogenized/concentrated limit (in a proper topology of convergence) yields 3 different diffusion processes for the second messengers cGMP and Ca^2+^. The first takes place in the interior of the limiting truncated cone Ω, free of its hindrances. The second occurs on the limiting activation fold, which is now a disc and cross-section of Ω with the plane *z* = *z*_*o*_, and the third occurs on the limiting sliver *S*. The various boundary conditions expressed in Eqs 4–9 are transformed into interior terms and link these otherwise three distinct diffusion processes. The pointwise form of such a limiting system is:

#### Homogenized interior limit

$$\begin{array}{c} \begin{array}{cc} {\text{[cG]}}_{t}-{\text{D}}_{\text{cG}}\Delta_{\bar{x}}\text{[cG]} & =-(\beta_{\text{dark}}\text{[cG]}-\alpha \left({\text{Ca}}^{2+}\right))\\ \text{[Ca}{}^{2+}{\text{]}}_{t}-{\text{D}}_{{\text{Ca}}^{2+}}\Delta_{\bar{x}}\text{[Ca}{}^{2+}\text{]} & =0 \end{array}\;\;\text{in}\;\;\Omega ; \end{array}$$ (13)

#### Homogenized limit in the limiting activated fold

$$\begin{array}{c} \begin{array}{cc} {\text{[cG]}}_{t}-{\text{D}}_{\text{cG}}\Delta_{\bar{x}}\text{[cG]} & =-(\beta_{\text{dark}}\text{[cG]}-\alpha \left({\text{Ca}}^{2+}\right))-\frac{1}{\nu \epsilon_{o}}{\text{k}}_{\sigma ;\text{hyd}}^{*}\text{[cG]}\left[{\text{E}}^{*}\right]\\ \text{[Ca}{}^{2+}{\text{]}}_{t}-{\text{D}}_{{\text{Ca}}^{2+}}\Delta_{\bar{x}}\text{[Ca}{}^{2+}\text{]} & =0 \end{array} \end{array}$$ (14)

#### Homogenized limit in the limiting sliver *S*

$$\begin{array}{c} \begin{array}{cc} {\text{[cG]}}_{t}-{\text{D}}_{\text{cG}}\Delta_{\text{S}}\text{[cG]} & =-\frac{1}{\epsilon_{o}\left(1+\nu \right)}\frac{\nu }{\sigma \cos\gamma }{\text{D}}_{\text{cG}}\nabla_{\bar{x}}\text{[cG]}\cdot \vec{n}-\delta_{z_{o}}\frac{\nu }{\sigma \cos\gamma }{\text{D}}_{\text{cG}}\nabla_{\bar{x}}\text{[cG]}\cdot \vec{e}\;\\ \text{[Ca}{}^{2+}{\text{]}}_{t}-{\text{D}}_{{\text{Ca}}^{2+}}\Delta_{\text{S}}\text{[Ca}{}^{2+}\text{]} & =-\frac{1}{\epsilon_{o}\left(1+\nu \right)}\frac{\nu }{\sigma \cos\gamma }{\text{D}}_{{\text{Ca}}^{2+}}\nabla_{\bar{x}}\text{[Ca}{}^{2+}\text{]}\cdot \vec{n}\\ & \;-\frac{1}{\epsilon_{o}\sigma \cos\gamma }\frac{1}{{\text{B}}_{{\text{Ca}}^{2+}}F}({\text{J}}_{\text{ex}}\left(\text{[Ca}{}^{2+}\text{]}\right)-\frac{1}{2}f_{{\text{Ca}}^{2+}}{\text{J}}_{\text{cG}}\left(\text{[cG]}\right))\\ & \;-\delta_{z_{o}}\frac{\nu }{\sigma \cos\gamma }{\text{D}}_{{\text{Ca}}^{2+}}\nabla_{\bar{x}}\text{[Ca}{}^{2+}\text{]}\cdot \vec{e}. \end{array} \end{array}$$ (15)

The various differential operators $\Delta_{\bar{x}}$ and $\nabla_{\bar{x}}$ act on the horizontal variables only, $\bar{x}=\left(x,y\right)$, and Δ_S_ is the Laplace-Beltrami diffusion operator on the limiting closing margin S. Also, $\vec{n}$ is the unit vector exterior to the truncated cone Ω, on the limiting sliver, whereas $\vec{e}$ is the unit vector exterior to the limiting activated disc at its intersection with the limiting sliver. The angle *γ* is the aperture of the right circular cone from which Ω has been truncated.

We examine briefly how the small scale geometry is expressed in the equations: in the interior volume of the cone, Eq 13 shows that the three dimensional space diffusion term of Eq 3 has been replaced by the two dimensional diffusion term $\Delta_{\bar{x}}=\partial^{2}/\partial_{x}^{2}+\partial^{2}/\partial_{y}^{2}$. The chambers of the COS may then be regarded as barriers to *z*-dimensional diffusion. Owing to their small thickness, homogenization shows the chambers effectively eliminating all diffusion in the vertical direction. The discs’ horizontal orientiation is remembered by the $\Delta_{\bar{x}}$ operator. At any activation chamber, Eq 14 shows that the thin volume there has been retracted onto a two-dimensional cross section where now the G-protein transduction machinery is expressed also. Finally, Eq 15 shows that the volume diffusion which took place within the closed margin has been transformed into a standard surface diffusion at the sliver, accordingly driven by the cone’s Laplace-Beltrami operator there. The calcium ion channels are also present here since the channels have been assumed to be located only at the sliver. The remaining flux terms quantify how the biophysics is coupled across all three spatial domain types: the interior, the activation site, and the sliver. These terms are formal in nature as, mathematically, the various concentrations [cG] and [Ca^2+^] in each of these equations represent, *a priori*, different unknown functions which must be simultaneously determined by the system. The system is also formal as the various functions involved might not have sufficient regularity to support the indicated, pointwise differential operations. Part of the theory includes showing that the values of [cG] and [Ca^2+^]—for example, in the sliver diffusion process Eq 15—are the same as the interior values when computed on the sliver (traces of [cG] and [Ca^2+^] on *S*). This consistent and mathematically rigorous interpretation of the system Eqs 13–15 is achieved through its weak formulation given in (S1 Appendix). Such a weak formulation is, in turn, the basis of the Matlab code. Homogenization and concentrated capacity do not affect the activation Eq 10, by either single or multiple isomerization, since such systems operate on (already concentrated) 2-dimensional domains.

### Longitudinal and well-stirred models

From the space-resolved nonhomogenized model in Eqs 3–8 one can derive a longitudinally well-stirred (LWS) model by interpreting all quantities as dependent on time *t* and the longitudinal variable *z* ∈ (0, *H*), along the axis of the cone, but independent of the horizontal variables $\bar{x}=\left(x,y\right)$. In addition one removes the hindrances due to the discs and regards all quantities as lumped on the axis of the cone while disregarding the geometry and lack of radial symmetry due to the presence of the closed margin. The boundary source terms in Eqs 7 and 8 are also lumped on the axis of the cone as sources interior to the segment (0, *H*). The governing equations for the diffusion of [cG] and [Ca^2+^] become

$$\begin{array}{c} \begin{array}{cc} {\text{[cG]}}_{t}-{\text{D}}_{\text{cG}}{\text{[cG]}}_{zz} & =-\eta \alpha \left(\text{[Ca}{}^{2+}\text{]}\right)-\eta \beta_{\text{dark}}\text{[cG]}-\delta_{z_{o}}{\text{k}}_{\text{hyd}}^{*}\left[{\text{E}}^{*}\right]\text{[cG]}\\ \text{[Ca}{}^{2+}{\text{]}}_{t}-{\text{D}}_{{\text{Ca}}^{2+}}\text{[Ca}{}^{2+}{\text{]}}_{zz} & =\frac{1}{{\text{B}}_{{\text{Ca}}^{2+}}F}(\frac{1}{2}f_{{\text{Ca}}^{2+}}{\text{J}}_{\text{cG}}\left(\text{[cG]}\right)-{\text{J}}_{\text{ex}}\left(\text{[Ca}{}^{2+}\text{]}\right)) \end{array}\;\;\text{in}\;\;\left(0,H\right) \end{array}$$ (16)

where ${\text{k}}_{\text{hyd}}^{*}$ is the *volumic* hydrolysis rate of cGMP by E* and $\delta_{z_{o}}$ is the longitudinal Dirac mass at *z*_*o*_. The activation mechanism is as in Eq 10 where diffusion is disregarded, the Brownian path *t* → **x**(*t*) is removed, the deactivation steps are lumped into a single one with catalytic activity *ν*_*o*_, and the locality of the activation site *z* = *z*_*o*_ is neglected. The governing equations become a system of ODE’s

$$\begin{array}{c} {\left[{\text{T}}^{*}\right]}_{t}-=\nu_{o}-{\text{k}}_{\text{T}{\text{E}}^{*}}\left[\text{E}\right]\left[{\text{T}}^{*}\right];\;{\left[{\text{E}}^{*}\right]}_{t}={\text{k}}_{\text{T}{\text{E}}^{*}}\left[\text{E}\right]\left[{\text{T}}^{*}\right]-{\text{k}}_{{\text{E}}^{*}}\left[{\text{E}}^{*}\right]. \end{array}$$ (17)

A globally well-stirred (GWS) model is derived from this by removing diffusion along the axis of the cone, thereby regarding the various quantities as independent of any geometry and lumped at a single point. Formally it follows from Eqs 16 and 17 by setting [cG]_*zz*_ = [Ca^2+^]_*zz*_ = 0.

#### The finite element code

Both the nonhomogenized and homogenized models have been implemented as separate finite element codes in Matlab and are freely available at [58]. In particular, the sections of their code that model second messenger diffusion were built independently for mutual validation. Maple was also used for the local element assembly. Maple produced the master coordinate representation of the PDE terms at each element. This output was then imported into Matlab. The finite element codes have been built like those described in [41] for rods. The key technical points, as well as differences between the cone HOM and NHOM cases, are summarized below.

Diffusion of G-protein transducer [T*] and PDE effector [E*] following activation is modeled by Eq 10, enforced at each activation disc. These equations are integrated through a standard Galerkin, spatial discretization over each of the cone’s cross sections that contain a site of photon isomerization. The cross sectional mesh is comprised by triangular elements leading to a continuous, piece-wise linear spline basis for [T*] and [E*]. The time integration is conducted by an implicit finite-difference scheme to guarantee numerical stability. The user specifies the isomerization site by supplying the code its z-levels and its horizontal location in polar coordinates. These coordinates define the dirac-mass source term used to generate R*. For purposes of showing agreement between HOM and NHOM, simulations in this paper assume that arrestin may shut off R*, independent of its phosphorlyated states. (It has been shown that rod arrestin-1 needs three receptor-attached phosphates to bind rhodopsin with high affinity [59]). Simulations have also assumed that the spatial location of R* and its quenching time by arrestin binding are both fixed. These assumptions are not restrictive towards showing agreement. Indeed, as remarked, the activation-deactivation mechanism in Eq 10 is not affected by the homogenized and concentrated limit, and its output served only as input common to the nonhomogenized model, through Eq 7, and the homogenized model through Eq 14. The code, however, has been written for the fully general model, including random shut-off of R* after a random number of phosphorylations.

The time to R* shut-off is deterministically taken as the mean sojourn time defined by Continuous Time Markov Chain for the rate of acquiring a phosphate λ_*o*_, and the rate *μ*_*o*_ of arrestin binding. This mean sojourn time is numerically computed in Matlab using the framework of [57]. Once the lifetime *τ*_R*_ of R* is fixed, one computes the diffusion of activated G-protein [T*] and activated PDE effector [E*] from Eq 10. The output [E*] is then used as boundary data in the volumic diffusion of cGMP and Ca^2+^.

Volumic diffusion of [cG] and [Ca^2+^] is computed by a system of partial differential equations coupled through their Neumann data. The nonhomogenized and homogenized codes are substantially different here. In the latter, the domain is a truncated, right, circular cone while in the nonhomogenized model it is a stack of conical chambers connected through a sliver. These two distinct meshes have been implemented in Matlab. Both are comprised of tetrahedral elements whose top and bottom faces have been scaled to the radius of the cone at their given heights. Here HOM has a significant performance advantage because it encodes the interdiscal chambers through the parameters *ν* and *ϵ*_*o*_ entered by the user into Eqs 13–15 rather than being explicit in the two-scale 3d geometry of NHOM.

Galerkin, spatial discretization is again used with shape functions determined by an isoparameteric mapping of a reference prism to each element in the respective NHOM and HOM cone meshes. The resulting nonlinear system is solved through an implicit finite-difference method for stability. This type of solver will also be required for future investigations that explore the calcium-dependent regulation of the phosphorylation of cone opsin by visinin [60].

HOM simulations were performed on a laptop with 4 GB of ram and a CPU with two cores at base frequency 1.60 GHz and max turbo frequency at 2.30 GHz. NHOM simulations were performed on the Ohio State Supercomputer Center’s Oakley Cluster [61].

#### A first choice of species parameters

Photoreceptor geometry alone does not drive the signature differences between rod and cone photoresponse [62, 63]. However, cone morphology is responsible for some relevant biophysical functions, even independent of the signaling cascade itself. For example, taper may reduce energetic costs of the COS and regulate noise [64].

There are significant differences in the photoreceptors’ respective biochemistries [2, 5]. To the best of our knowledge no complete and measured parameter set for cone biochemistry yet exists for any one species. For rod parameters see [31], for example. For this paper, measured parameters were taken from the literature where possible. Occasionally, the only values found were from different species (Table 1). As a consequence the presented numerical simulations cannot correspond to any single species. Even among reported parameters, several were fit using models and were not from experiment. Where parameters could not be found at all, estimates were attempted by using known concentration differences in rods and cones and then scaling the reported rod values in [31]. For these reasons, the parameters which populate these simulations are neither exhaustive nor definitive. They are used here only to present the numerical agreement between the HOM and NHOM models. Once validated, the HOM model, in view of its speed of execution, can be used to perform almost real time virtual experiments to refine parameters and analyse their sensitivity. The parameters used in the simulations are collected in Table 1. Their choice is explained in (S1 Appendix).

**Table 1:** Adopted parameters for comparing COS model types.

Symbol	Units	Definition	Species	Value	Reference
*α*_max_	*μMs*^−1^	Maximum rate of *cGMP* synthesis at high Ca^2+^ concentration		1311	[31, 66]
*α*_max_/*α*_min_	-	Suppression ratio of *α* from high to low Ca^2+^ concentration		13.9	[31]
*β*_dark_	*s*^−1^	Rate of cGMP hydrolysis by dark activated PDE		67	Computed
	*s*^−1^		Carp	∼ rod	[30]
*B*_cG_	-	Buffering Power of Cytoplasm for cGMP		1	[31, 67, 68]
*B*_Ca_	-	Buffering Power of Cytoplasm for Ca^2+^	Striped Bass	20	[5, 68]
*c*_*TE*_	-	Coupling Coefficient from G* to E*		1	[31]
[cG]_dark_	*μM*	Concentration of cGMP in the dark	Carp	2	[66]
			Striped Bass	27.9 ± 14.9	[5]
[Ca^2+^]_dark_	*μM*	Concentration of Ca^2+^ in the dark	Striped Bass	.4	[5]
			Salamander	.41	[69, 70]
*r*_base_	*μm*	Radius of COS base	Striped Bass	3.08 ± .31	[12]
			Tiger Salamander	2.5, 2	[1, 12]
			Human	1.5	[12]
			Turtle	1.25	[1]
			Primate	1.5	[1]
*r*_tip_	*μm*	Radius of COS tip	Striped Bass	1.15 ± .15	[12]
			Tiger Salamander	1.1, 1.25	[1, 12]
			Human	.75	[12]
			Turtle	.5	[1]
			Primate	.5	[1]
*ω*_0_	-	Open margin angle for sliver	Striped Bass	*π*	[12]
			Frog	*π*	[11]
D_cG_	*μm*^2^ *s*^−1^	Diffusion Coefficient for cGMP	Mouse Rod	120	[31]
D_Ca_	*μm*^2^ *s*^−1^	Diffusion Coefficient for Ca^2+^	Mouse Rod	15	[31]
D_E_	*μm*^2^ *s*^−1^	Diffusion Coefficient for activated PDE	Mouse Rod	1.2	[31]
D_T_	*μm*^2^ *s*^−1^	Diffusion Coefficient for activated G-protein	Mouse Rod	2.2	[31]
D_R_	*μm*^2^ *s*^−1^	Diffusion Coefficient for activated Opsin	Mouse Rod	1.5	[31]
*ϵ*	nm	Disc thickness	Striped Bass	15	[12]
*η*	nm	Volume to surface ratio	Striped Bass	7.5	Computed
F	C/mol	Faraday’s constant		96 500	[31]
*f*_Ca_	-	Fraction of current carried by Ca^2+^	Striped Bass	.33 ± .08	[55, 68]
*H*	*μm*	Length of COS	Striped Bass	15.2 ± 1.46, 17	[12, 68]
			Tiger Salamander	8	[1]
			Turtle	15	[1]
			Primate	13	[1]
			Human	7	[12]
*J*_dark_	pA	Dark current	Striped Bass	27.3 ± 10.5	[5, 68]
			Tiger Salamander	50	[1]
			Primate	40	[1]
$J_{\text{cG}}^{\text{max}}$	pA	Maximum cGMP gated channel current (when saturate by cGMP)		2 500	[56, 68]
$J_{\text{ex}}^{\text{sat}}$	pA	Saturated exchanger current		4.87 ± 1.88	[68]
*k*_cat_/*K*_*m*_	*μM*^−1^ *s*^−1^	Hydrolytic efficiency of activated PDE dimer	Striped Bass	500	[71]
*k*_*σ*,hyd_	*μm*^3^ *s*^−1^	Surface hydrolysis rate of cGMP by dark-activated PDE		5.02 * 10^−4^	Computed
$k_{\sigma ,\text{hyd}}^{*}$	*μm*^3^ *s*^−1^	Surface hydrolysis rate of cGMP by light-activated PDE		.83	Computed
*k*_*E*_	*s*^−1^	Rate constant for inactivation of PDE	Striped Bass	18.5	[71]
*n*_step_	-	Number of phosphorylation states used in CTMC	*	1	Numerical
λ_0_	*s*^−1^	Initial rate of phosphorylation in CTMC	*	105	Estimated
*μ*_0_	*s*^−1^	Rate of arrestin binding in CTMC	*	12.5	Estimated
*k*_*v*_	-	Decay constant of phosphorylated opsin’s catalytic activity in CTMC	Mouse Rod	.5	[57]
*K*_cyc_	*nM*	Half-saturation [Ca^2+^] for GC activity	Striped Bass	100	[68]
*K*_cG_	*μM*	[cGMP] for half-maximum cGMP-gated channel opening	Mouse Rod	20	[31]
*K*_ex_	*μM*	[Ca^2+^] for half-maximum exchanger channel opening	*	.69	Numerical
*ν*	-	Ratio between interdiscal space and disc thickness		1	Computed
*ν*_*ϵ*_	nm	Interdiscal space thickness	Striped Bass	15	[12]
*ν*_*RE*_	*s*^−1^	Rate of PDE formation per fully activated Rh	Striped Bass	125	[71]
			Carp	30	[67, 72]
*ν*_*RG*_	*s*^−1^	Rate of Transducin formation per fully activated Rh	Striped Bass	125	Computed
			Carp	30, 33	[67, 72]
*n*	-	Number of discs		500	Computed
*N*_AV_	mol^−1^	Avogadro Number		6.02 * 10^23^	[31]
*m*_cyc_	-	Hill coefficient for GC effect	Mouse Rod	2.5	[31]
			Striped Bass	2	[68]
*m*_cG_	-	Hill coefficient for cGMP-gated channel	Striped Bass	2.5	[56, 68, 73]
[PDE]_*σ*_	*μm*^−2^	Surface density of dark activated PDE	Mouse Rod	1000	[31]
*σ*	-	Ratio between the disc thickness and sliver thickness	Striped Bass	1	[12]
*σ*_*ϵ*_	nm	Distance between the disc rim and outer plasma membrane at sliver	Striped Bass	15	[12]

Parameters are used to populate NHOM, HOM, LWS, GWS simulations, and validate model types.

### Performed simulations and datasets

Numerical simulations of the COS, finite element system were performed in MATLAB. The resulting HOM and NHOM datasets have been submitted to the Dryad repository [65]. The simulations use the set of parameters in Table 1, whose choices are discussed in (S1 Appendix). Three minor exceptions are the parameters [cG]_dark_, [Ca^2+^]_dark_ and *j*_dark_ whose values are actually determined by mass balance principles and other model parameters (S1 Appendix).

## Results and discussion

**Fig 2:**
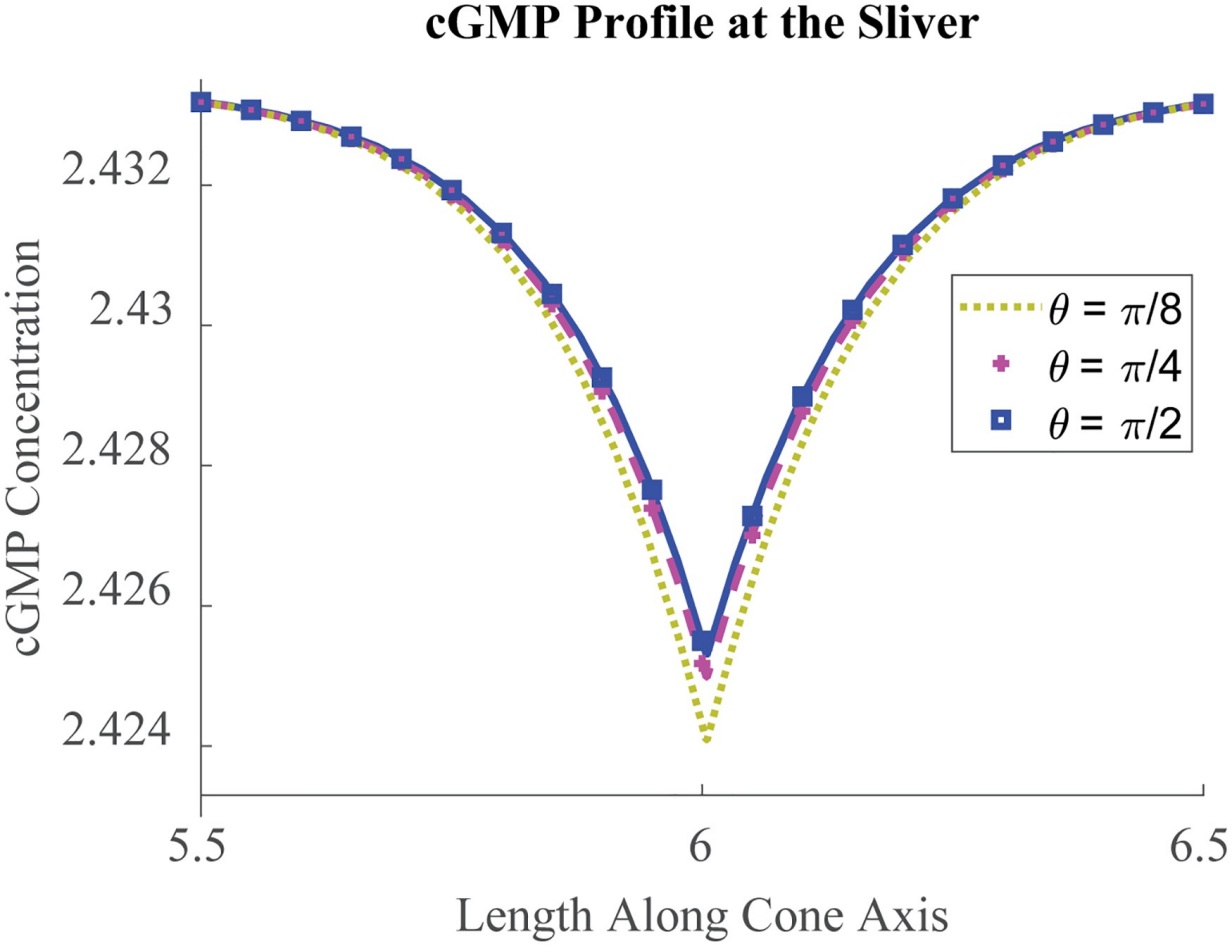
cGMP profile at the sliver. [cG](*z*, *θ*, t_peak_) (*μM*) profile shown at the sliver for several samples of *θ* after photopigment isomerization at the disc center. Here *z* ∈ (0, *H*) indicates height level (*μm*) and *θ* ∈ (0, *π*) spans the sliver, *ie* closed margin. From its asymmetrical attachment, the sliver’s [cG] profile is shown to deplete more near the transition between margin types. This may be due to the transitions’ being closer to activated E*in the furthest parts of the disc than, for example, is the sliver’s center. The profile is still symmetric about the sliver’s center. One computes the signal spread to be 0.31 *μm*, where signal spread is reported as the length of interval in which cGMP drop is greater than 1/*e* times the peak drop [12, 76]. Note [41] uses an alternative measure of spread to better appreciate the spread’s time evolution.

Numerical experiments are performed for ten and single photon response (respectively TPR, SPR). For TPR ten photons are placed at the center of ten equispaced, middle discs, while for SPR a single photon is placed at the center of the middle disc. SPR is not experimentally detectable in native cones because of underlying noise from spontaneous thermal activation of cone pigment [74, 75]. Cone photoreceptors are capable of signaling above noise with 4-12 photons [68]. However, SPR may be modeled for a virtual, noise-free cone, to investigate the phototransduction functions independent of spontaneous thermal activation. As an example, for a single isomerization placed at the center of the activated disc, Fig 2 shows the fully spatially resolved model’s noise-independent prediction for the cGMP profile at the sliver, where the channels are located, at the time t_peak_ of peak current suppression.

**Fig 3:**
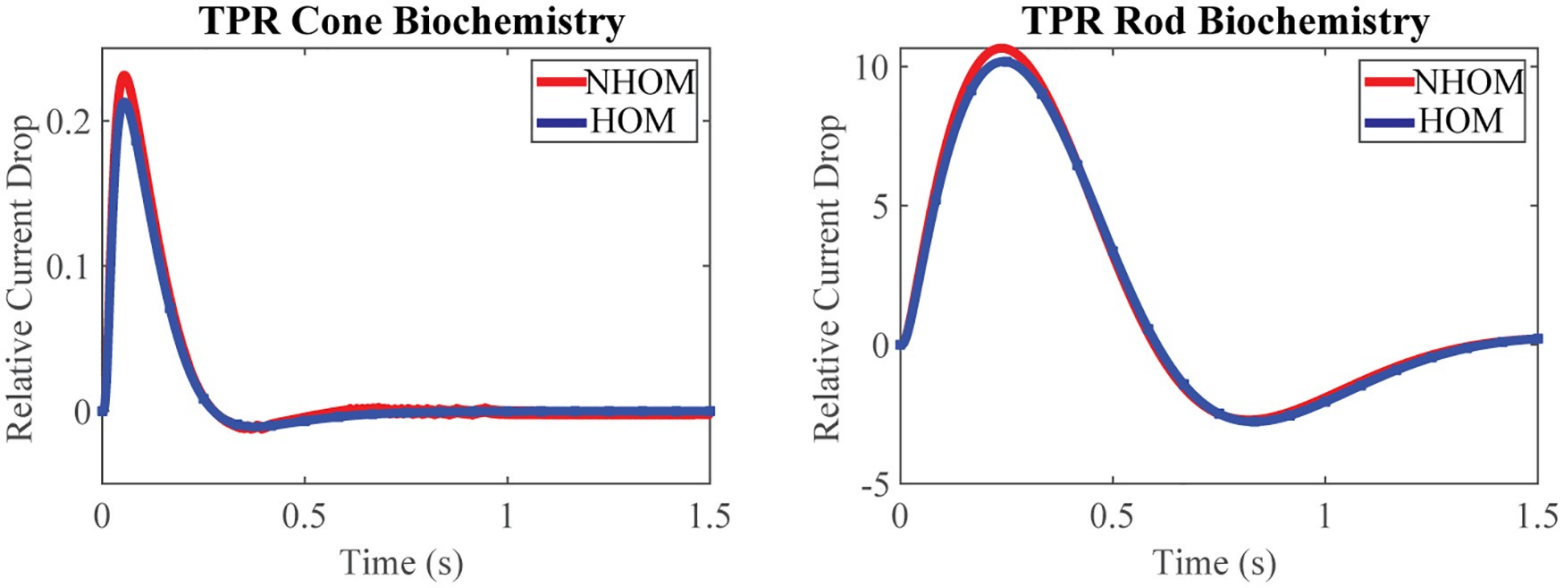
TPR response varying biochemistries. Relative current drop (units %) for ten photon response (TPR) as predicted by NHOM (red) and HOM (blue). The x-axis is the time after photon detection, and the y-axis is the relative drop in current as a percentage of the dark current: 100(*j*_dark_ − *j*(*t*))/*j*_dark_. Photon isomerization occurred at disc center 40% of the height above the larger cone base up to 58% of the height. Both panels exhibit cone morphology with ion channels localized to the sliver. However, the left panel presents cone biochemistry (Table 1), while the right panel presents rod biochemistry [31]. The relative error in current drop is ≈ 4.5% for the right panel and rises to ≈ 7.99% for the left panel. This may be due to biochemical differences and not represent a numerical artifact (S1 Appendix). The TPR response is scaled by a factor ≈ 10 compared to the SPR (not shown) but exhibits the same characteristics. This finding is consistent with [47], but is not expected to hold as light intensity increases. Undershoot in cone response has been observed in several species [46, 47, 68], although its prevalence is still debated [48]. Though slight in the case of cone biochemistry due to dim light, undershoot is observed in both panels. This is striking since mouse rod biochemistry in mouse rod morphology exhibits no undershoot (Fig 5 in [31]). This evidences that OS morphology and ion channel localization contribute to the flash response.

In addition to the native cone response, rod biochemistry expressed on cone photoreceptor geometry and its response are also shown. Some experimental attempts to realize such hybrids are in [77] (cone PDE into ROS), with the purpose of separating the role of the biochemistry from that of geometry and exploring how each influences the photoresponse when the other is unchanged. The homogenized model can virtually separate the biochemical basis of photoresponse from the 3d geometry, a feature not accessible to existing well-stirred and 1D models [5, 12, 47, 78–80]. In the rod biochemistry panel of Fig 3, the parameters are that of mouse ROS and taken from [31]. In the cone biochemistry panel of Fig 3, the biochemistry is given by Table 1. The simulations show that the COS’ 3D geometry and ion channel localization at the sliver can contribute to undershoot. This is striking since undershoot is not observed with the mouse rod morphology and biochemistry of [31]. Only the mouse SPR is shown there. However the SPR counterpart of Fig 3 exhibits the same features, except it is scaled by a factor ∼1/10 (not shown). Further, the GWS and LWS models which lack 3D spatial resolution, presented in a later section, do not exhibit undershoot.

While parameter analysis is not the aim of this paper, the selected parameters of cone photoreceptors yield responses with similarities to those experimentally observed in the literature. For example [26] reports t_peak_ ≈ 70ms in mouse cone, and [48] reports t_peak_ ≈ 40ms in primate cones after applying a low-pass, signal filter. The TPR, 500-chamber homogenized simulation in Fig 3 yields t_peak_ = 56.7ms. Simulation parameters lead to a value *j*_dark_ = 14.95 *pA* which is near the lower range reported in [68]. At low light levels [47] finds that current suppression increases linearly with the number of isomerizations. This behavior is reproduced in simulation: Comparing TPR trials’ peak relative drop (Fig 3) with SPR peak relative drop (not shown), it is found they differ by a factor 0.21/0.019 = 11.05. One possible dissimilarity is that these parameters in HOM predict an SPR peak magnitude current drop of ≈ 0.003 pA when a photon is detected at the center of a disc four-tenths of the height above the COS’ larger radius. This is the location where most of the simulations presented in this paper are centered. This peak current drop is less than that extrapolated for the cone SPR by other authors, for example ≈ 0.03 pA in macaque [47] and ≈ 0.14 pA in striped bass [71]. However, HOM’s response-magnitude varies according to the 3D locations at which the photons are detected. For example, with Table 1’s parameters HOM predicts that the SPR for a photon detected 1 *μm* below the COS tip and one-eighth the radius away from the center of the sliver’s channels exhibits a peak magnitude drop ≈ 0.01 pA, more similar to that extrapolated in [47]. These numerical findings support the view that cones do not exhibit single photon response due to their drop not reaching a detectable magnitude above noise.

Finally, several retinal disorders, such as stationary night blindness and retinal degeneration, are known to be linked to missense mutations that lead to the loss of inhibition of PDE6 in rods by its regulatory subunits PDE6*γ* [81–83]. To test whether the model with these parameters would reproduce these findings in a cone geometry, dark activity of PDE was incrementally increased, and TPR was simulated (S1 Appendix). Sensitivity to light, measured as peak relative drop, decreased by a factor 4.63 as *β*_dark_ increased up to 150% of its value in Table 1. As expected, an increase in the basal activity of PDE was numerically predicted to desensitize the photoreceptor to dim light events.

### Numerical convergence of the nonhomogenized model to the homogenized model

The agreement in Fig 3 is remarkable with relative errors in current prediction (nonhomogenized versus homogenized) less than 0.49% at time *t* = t_peak_ for both biochemistries. However, the relative error of the current suppression, while of the order of 4.5% for the mouse ROS biochemistry on cone geometry, rises to about 7.99%, for the COS biochemistry on the cone geometry. Thus it would appear that the homogenized model with cone biochemistry less faithfully reproduces the nonhomogenized one than when both models express mouse ROS biochemistry. However the relative error in drop is rescaled by the nonhomogenized drop value J_dark_ − J_NHOM_, which in cones is significantly smaller than in rods. It follows that the observed differences in relative error are actually driven by the biochemical differences between rods and cones and not numerical artifact (S1 Appendix).

**Fig 4:**
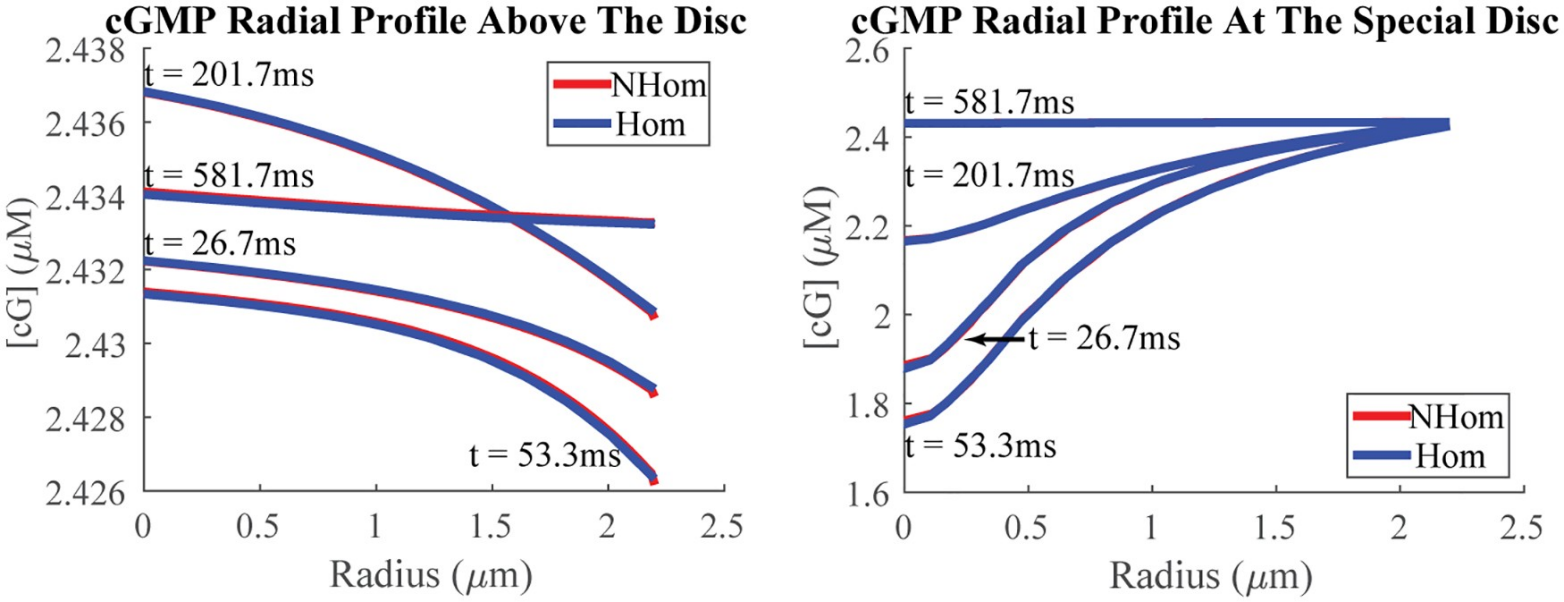
cGMP radial profiles. Shown is the agreement between radial profiles for cGMP above, and at a special disc as predicted by the nonhomogenized (red) and homogenized (blue) models at various times over transduction. The average relative error across sample points is less than 0.05% for all time points shown. These profiles have been taken starting from the center of the cone and moving radially towards the center of the sliver, which spans the angle range *θ* ∈ (0, *π*). The negative slope above the special disc is consistent with cGMP loss at these levels being due to signal spread through the sliver and originating from the special disc. The positive slope in the special disc is consistent with cGMP depletion being strongest at the R*, placed at the disc’s center. Recovery of cGMP back to baseline in the special disc is due to the shutting-off of R* and resynthesis by guanylate cyclase. The small appreciation of cGMP loss in the chamber above and separated from the special disc suggests photon detections in neighboring chambers are independent events in dim light. The change in cGMP is also smaller at the presumed site of ion channel localization, the sliver. There cGMP moves from a confined, horizontal chamber to a site where it can spread horizontally and vertically. Depletion is better compensated by cGMP diffusion. cGMP loss at the ion channels is mitigated, and 3D localization of ion channels with proximity to guanylate cyclase contributes to an undetectable cone SPR. The cGMP profile below the special disc is not shown due to its similarity with that above the special disc.

**Fig 5:**
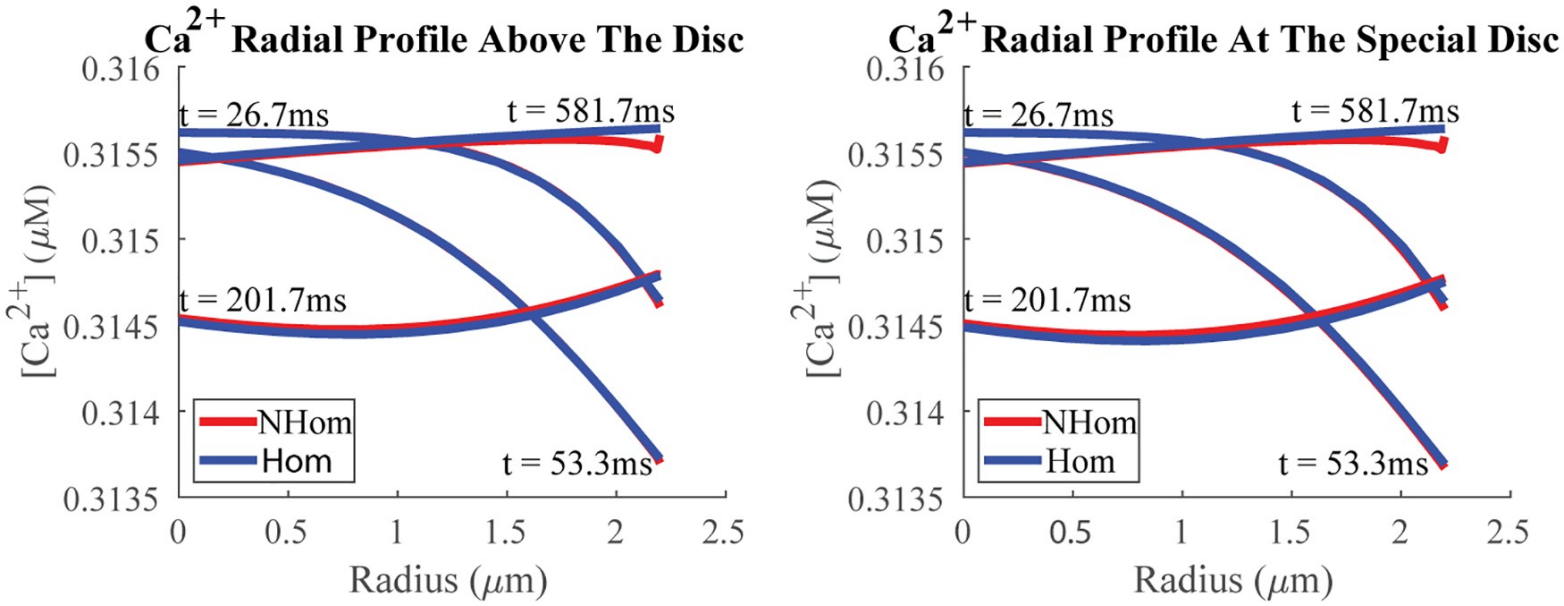
Ca^2+^ radial profiles. Shown is agreement between radial profiles for Ca^2+^ above and at an activation disc, where photopigment isomerization has occurred, as predicted by the nonhomogenized (red) and homogenized (blue) models at various times over transduction. The average relative error across sample points is less than 0.03% for all time points shown. The profile is taken towards the center of sliver, which spans the angle range *θ* ∈ (0, *π*). Ca^2+^ flux occurs at the sliver, the site of the channels. Changes in Ca^2+^ are driven by extrusion/intrusion there and not at the site of activated opsin. (Contrast this finding with Fig 4.) Small absolute changes in Ca^2+^ demonstrate another mechanism contributing to an undetectable SPR in cones. The Ca^2+^ profile below the special disc is not shown due to its similarity with those already included.

A remarkable spatial agreement between NHOM’s and HOM’s predicted cGMP and Ca^2+^ profiles is depicted in Figs 4 and 5. These figures show the radial profiles below, at, and above the activation disc at the angle *θ* = 0 (i.e., at the middle of the closing margin/sliver), for both the homogenized (blue) and nonhomogenized (red) models over several time points.

### Comparison of computational cost between HOM and NHOM

Table 2 reports the computational cost of running the NHOM model for the reported SPR and TPR trials on the Oakley Cluster at the Ohio Supercomputer Center (OSC). Phototransduction was simulated for 1.5*s* with 900 time steps and near 7 million degrees of freedom for the cGMP and Ca^2+^ system of NHOM. Conversely, the HOM model compared to NHOM in Figs 3, 4 and 5 routinely ran in a few minutes on a laptop.

**Table 2:** Time and memory costs reported by the log files of the Ohio Supercomputer Center (OSC) in time integrating a standard implementation of the NHOM model. NHOM results are those shown in Figs 3, 4 and 5. Total memory (column 5) is the sum of physical and virtual memory (columns 3 and 4). Computational cost of space integration is not shown. Conversely, the HOM model ran the same, entire SPR and TPR trials in a matter of minutes on a laptop.

NHOM COST	CPU TIME	MEM	VMEM	TOT MEM	SOL FILE SIZE
SPR	20.33 hr	104.71 GB	124.95 GB	229.66 GB	32.04 GB
TPR	17.30 hr	104.94 GB	125.61 GB	230.55 GB	24.74 GB

The NHOM code was implemented in a standard way, and no special measures were taken to optimize it for parallel computing. The costs reported reflect the time integration of the NHOM model and do not include the cost of assembling the mass and stiffness matrices over the geometry.

### Comparison with longitudinal and well-stirred models

**Fig 6:**
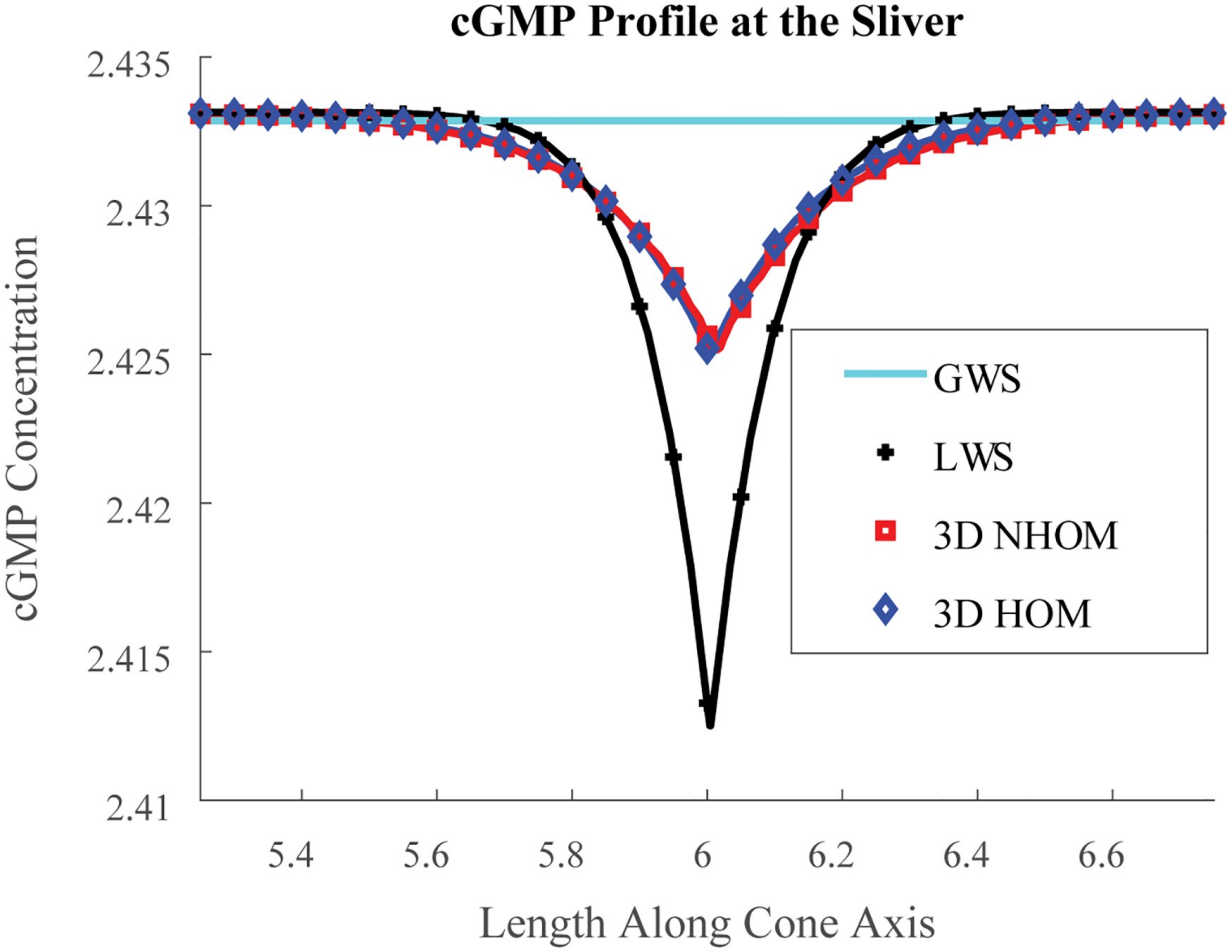
Profile at maximum current drop. [*cG*] (*μM*) profile shown at the instant of maximum current drop at the sliver’s center. The x-axis is along the cone length (*μm*) about the site of photon isomerization. All model types were populated with the parameters from Table 1. The homogeneity of the GWS model necessarily leads to an underestimation of the NHOM cGMP dynamics at the sliver. Conversely, the LWS model exaggerates the NHOM local depletion of cGMP at the site of photon isomerization.

The LWS and GWS models have been implemented and compared to both fully space-resolved homogenized and nonhomogenized models. Fig 6 shows the *z* → [cG](*z*, t_peak_) profile at peak time t_peak_, at the center of the closed margin (*θ* = 0), where the channels are placed. These profiles are those predicted by the HOM and NHOM fully space resolved models and also the LWS and GWS models. The simulations show that the largest cGMP suppression occurs for the LWS model. The latter indeed presupposes that the channels are all lumped at one point. The HOM model instead distributes them on the whole COS. The GWS produces a [cG] which, while varying in time, is constant in the space variable and, hence, is insensitive to the channel location.

**Fig 7:**
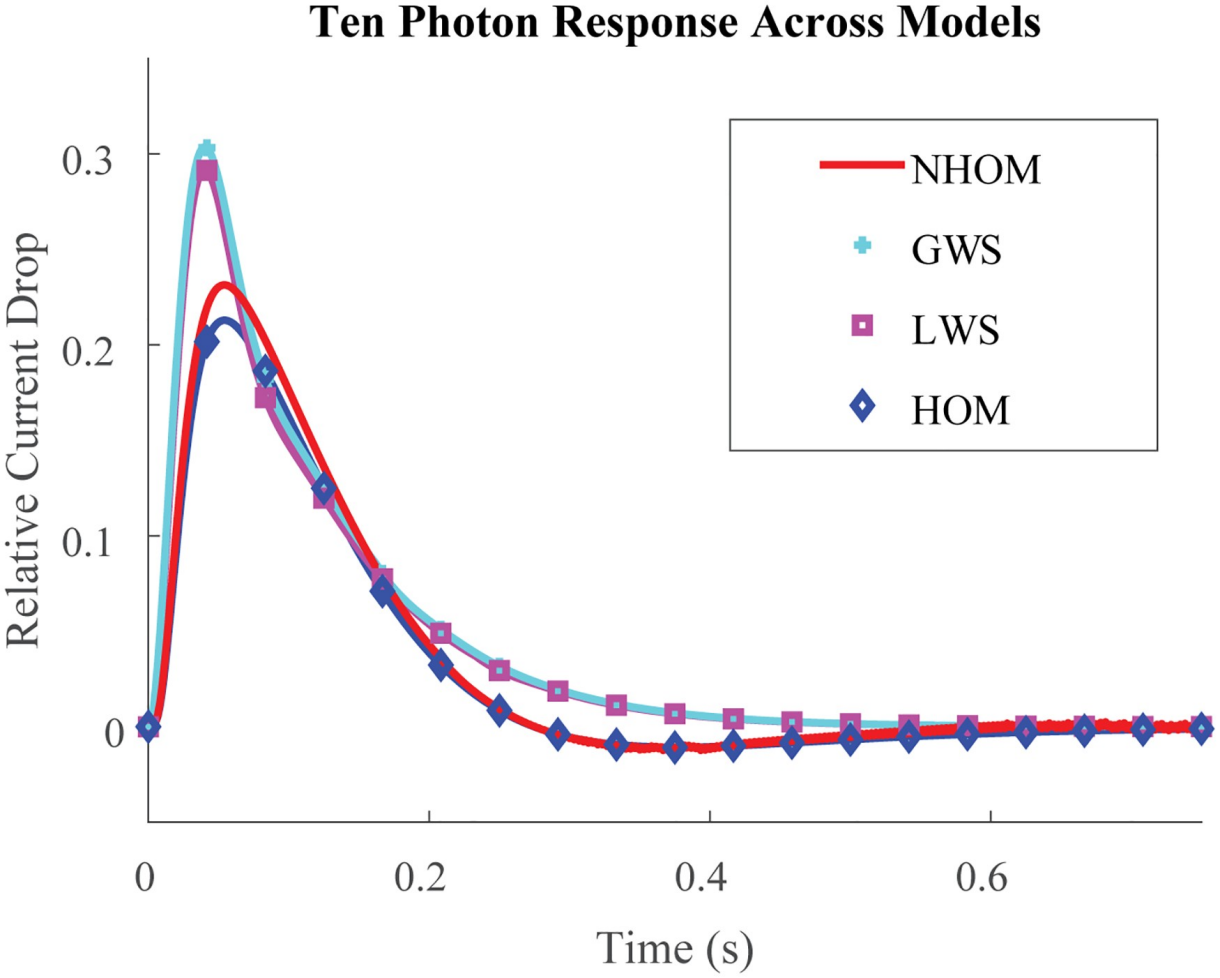
Ten photon response across models. Relative Current Drop (units %) for a ten photon isomerization event spanning from 40% of the length of the cone axis from bottom up to 58% of its length towards the top. The isomerizations are evenly spaced along the length and taken to occur at the center of each of the ten discs. The LWS and GWS models do not show the slight undershoot with these parameter values, suggesting that undershoot is influenced by 3D spatial effects. LWS and GWS have the time to peak shift left and occur ≈ 10 *ms* earlier than the 3D resolved models’. Thus reducing the space resolution of the models changes the time kinetics and exaggerates the response by suppressing the damping mechanisms due to diffusion.

Altogether Figs 4, 5, 3, 6 and 7 show how HOM faithfully reproduces the predictions of NHOM much more accurately than lesser space resolved models. Numerical trials show that LWS and GWS do not adequately describe the spatio-temporal evolution of cone signaling: Figs 6 and 7. These underscore the importance of geometry and its effects to the function of phototransduction. In particular, the 3D resolved models with ion channels localized to the sliver detected undershoot in the drop response while GWS and LWS did not. Moreover, HOM is computationally efficient and requires only a few minutes on a laptop to execute. Meanwhile, the standard, 3D resolved NHOM is much more expensive: Table 2. This reduction in cost is made possible through the mathematical theory of homogenization [36, 37]. For these reasons, the homogenized model is better suited for parameter analysis and estimation in cone photoreceptors.

## Supporting information

S1 Appendix Supplementary materials. In particular, this appendix includes the weak formulations for the homogenized model as well as the reasoning behind this particular choice of parameters.
(PDF)
