# Supplementary material for: Multi-scale, numerical modeling of spatio-temporal signaling in cone phototransduction
Source: PLoS One. 2019 Jul 25;14(7):e0219848. doi: 10.1371/journal.pone.0219848 (PMC6657853; doi:10.1371/journal.pone.0219848)
Supplement: S1 Appendix — In particular, this appendix includes the weak formulations for the homogenized model as well as the reasoning behind this particular choice of parameters. (PDF) [file pone.0219848.s001.pdf]

Supplement to multi-scale, numerical modeling of spatio-temporal signaling in cone phototransduction

C. Klaus, G. Caruso, V.V. Gurevich, and E. DiBenedetto

June 24, 2019

1 The Homogenized Model in Weak Form

The homogenized weak form for cGMP diffusion is given by

$$0 = \left(\frac{\nu}{1+\nu}\right)\left\{\int_{\Omega_T}\Big(-\varphi_t[\text{cG}] + D_{cG}\nabla_{\bar{x}}\varphi\nabla_{\bar{x}}[\text{cG}] + \left(\beta_{\text{dark}}[\text{cG}] - \alpha\left([\text{Ca}^{2+}]\right)\right)\varphi\Big)dxdt - \int_{\Omega}\varphi(\cdot,0)[\text{cG}]_0 dx\right\}$$
$$+\nu\epsilon_0\left\{\int_{D_{z_0,T}}\Big(-\varphi_t[\bar{\text{cG}}] + D_{cG}\nabla_{\bar{x}}\varphi\nabla_{\bar{x}}[\bar{\text{cG}}] + \Big[\Big(\beta_{\text{dark}}[\bar{\text{cG}}] - \alpha\left([\bar{\text{Ca}}^{2+}]\right)\Big) + \frac{1}{\nu\epsilon_0}[\bar{\text{cG}}]f_1\left([\bar{\text{Ca}}^{2+}]\right)\Big]\varphi\Big)d\bar{x}dt\right.$$
$$\left. - \int_{D_{z_0}}\varphi(\cdot,0)[\text{cG}]_0 d\bar{x}\right\}$$
$$+\sigma\epsilon_0\cos(\gamma)\left\{\int_{S_T}\Big(-\varphi_t[\hat{\text{cG}}] + D_{cG}\nabla_S\varphi\cdot\nabla_S[\hat{\text{cG}}]\Big)dSdt - \int_{\partial\Omega_S}\varphi(\cdot,0)[\text{cG}]_0 dS\right\} \quad (1)$$

Here, where $[\text{E}^*]$ is generated by the G-protein activation cascade,

$$f_1\left([\bar{\text{Ca}}^{2+}]\right) = k^*_{\sigma,\text{hyd}}[\text{E}^*] \quad (2)$$

The homogenized weak form for Ca^{2+} diffusion is given by

$$0 = \left(\frac{\nu}{1+\nu}\right)\left\{\int_{\Omega_T}\Big(-\varphi_t[\text{Ca}^{2+}] + D_{Ca}\nabla_{\bar{x}}\varphi\nabla_{\bar{x}}[\text{Ca}^{2+}]\Big)dxdt - \int_{\Omega}\varphi(\cdot,0)[\text{Ca}^{2+}]_0 dx\right\}$$
$$+\nu\epsilon_0\left\{\int_{D_{z_0,T}}\Big(-\varphi_t[\bar{\text{Ca}}^{2+}] + D_{Ca}\nabla_{\bar{x}}\varphi\nabla_{\bar{x}}[\bar{\text{Ca}}^{2+}]\Big) - \int_{D_{z_0}}\varphi(\cdot,0)[\text{Ca}^{2+}]_0 d\bar{x}\right\}$$
$$+\sigma\epsilon_0\cos(\gamma)\left\{\int_{S_T}\Big(-\varphi_t[\hat{\text{Ca}}^{2+}] + D_{Ca}\nabla_S\varphi\cdot\nabla_S[\hat{\text{Ca}}^{2+}] + \frac{1}{\sigma\epsilon_0\cos(\gamma)}\Big(g_1\left([\hat{\text{Ca}}^{2+}]\right) - g_2\left([\hat{\text{cG}}]\right)\Big)\varphi\Big)dSdt\right.$$
$$\left. - \int_{\partial\Omega_S}\varphi(\cdot,0)[\text{Ca}^{2+}]_0 dS\right\} \quad (3)$$

Here

$$\int_S \nabla_S\varphi\cdot\nabla_S\hat{w}dS = \int_{\theta=-\omega_0}^{\omega_0}\int_{z=0}^{H}\left(\frac{1}{[\lambda(z)]^2 r^2}\varphi_\theta\hat{w}_\theta + \cos^2(\gamma)\varphi_z\hat{w}_z\right)\lambda(z)r\sec(\gamma)d\theta dz \quad (4)$$

while also

$$g_1\left([\text{Ca}^{2+}]\right) = \frac{1}{B_{\text{Ca}^{2+}}\mathcal{F}}\text{J}_{\text{ex}}^{\text{sat}}\left([\text{Ca}^{2+}]\right) \quad \text{and} \quad g_2\left([\text{cG}]\right) = -\frac{1}{2}\frac{1}{B_{\text{Ca}^{2+}}\mathcal{F}}f_{\text{Ca}^{2+}}\text{J}_{\text{cG}}^{\text{max}}\left([\text{cG}]\right) \quad (5)$$

Note that $\text{J}_{\text{ex}}^{\text{sat}}, \text{J}_{\text{cG}}^{\text{max}}$ are defined in (7), that from (4) the principal part in the sliver is a Laplace-Beltrami driven scaled by the normal width of the sliver, and that the test function class consists of all φ which are C^∞ smooth in the space-time variables.

2 Choice of Model Parameters

2.0.1 GC Activity.

The synthesis rate of cGMP follows a spatially localized Hill-type law owing to the binding of Ca^{2+} to GCAPS:

$$\alpha\left([\text{Ca}^{2+}]\right) = \alpha_{\min} + \frac{\alpha_{\max} - \alpha_{\min}}{1 + \left([\text{Ca}^{2+}]/K_{cyc}\right)^{m_{cyc}}} \tag{6}$$

The quantities $\alpha_{\min}$ and $\alpha_{\max}$ are respectively the least and greatest rate of cGMP synthesis by GC. m_{cyc} is the Hill coefficient, and K_{cyc} is the concentration for the half-maximal rate. To estimate $\alpha_{\max}$, the concentration of guanylate cyclase in carp cones and carp rods respectively has been experimentally measured in (1) to be $72\mu M$ and $4.2\mu M$ respectively. Their ratio is then used to scale the (2) reported mouse rod value of $\alpha_{\max} = 76.5\mu M s^{-1}$. The value of $\alpha_{\max}/\alpha_{\min}$ is taken from mouse in (2).

2.0.2 Dark Hydrolysis.

The synthesis rate of cGMP in the dark is proportional with the dark concentration of cGMP through the proportionality constant β_{dark}. The value $\beta_{\text{dark}} = 67s^{-1}$ was chosen and used in models by appealing to mass balance principles. It achieved equilibrium balance between PDE hydrolysis and cyclase synthesis for the given choice of other model parameters:

$$\alpha_{\min} + \frac{\alpha_{\max} - \alpha_{\min}}{1 + ([Ca^{2+}]_{\text{dark}}/K_{cyc})^{m_{\text{cyc}}}} = \beta_{\text{dark}}[\text{cG}]_{\text{dark}}$$

2.0.3 Buffers:

For B_{cG}, in (3) the total quantities of PDE in carp cone and rods were reported as similar, so that their ratio is taken as 1. The buffering power for cGMP has been estimated as that ratio, 1, times the mouse rod value $B_{cG} = 1$ reported in (2). For B_{Ca}, (4) argues that the calcium buffer mechanism is more complicated in cones and varies as a function of calcium. Text equation (1.9) and Table 3 there propose a model and parameterize this dependence. At dark concentrations, $[\text{Ca}^{2+}]_{\text{dark}} = 0.4\mu M$, which leads to an estimate $B_{Ca} = 20.01$. This is consistent with (2)'s value of 20 in mouse rod.

2.0.4 Coupling Coefficient

The value c_{TE} is the ratio ν_{RE}/ν_{RG}. The mouse rod value, 1, in (2) is taken.

2.0.5 Dark Steady State.

The concentration of cGMP in the dark was estimated in Carp (1) while measured by a model from samples of experimental dark current values in Striped Bass (4, 5). Calcium concentration in the dark was reported for striped bass in (4) and for Salamander in (6, 7). The dark values of cGMP and Ca are theoretically linked through a mass-balance argument. These fluxes encode the dark hydrolysis and resynthesis of cGMP as well as the balance of exchanger and cGMP-gated currents. In the dark, they are balanced. The reported values are used as initial guesses in a bisection method search for numerical solutions of the flux balance. This returned the simulation values of $[\text{cG}]_{\text{dark}} = 2.43\mu M$ and $[\text{Ca}^{2+}]_{\text{dark}} = 0.31\mu M$. These concentrations obtained by flux balance are then used as the true starting dark values.

2.0.6 Diffusion Coefficients

The values $D_{cG} - D_R$ are taken as they are for rods in (2).

2.0.7 Volume to Surface Ratio

η is the asymptotic conversion ratio to pass from the volumic density defined in a chamber and the surface area densities defined on discal faces (2):

$$\eta = \frac{\pi r_z^2 \nu \epsilon_0}{2\pi r_z^2} = \frac{1}{2}\nu\epsilon_0$$

2.0.8 Fraction of Current Carried by Ca^{2+}

(8) has found the fraction of current carried by calcium in cones to be .33 and larger than the .06 value reported for rods (2).

2.0.9 Circulating Dark Current

In the homogenized model, dark current is not a free parameter but is determined by other parameter choices. Present simulations report a value $J_{\text{dark}} = 14.95pA$. (See the discussion of K_{ex}).

2.0.10 Maximum cGMP-gated Current and Exchanger Current

The functional form of the currents is given by local Michaelis-Menten and Hill Laws (9). The exchanger current density and cGMP-gated channel current density are given respectively by

$$\mathrm{J}_{\text{ex}}^{\text{sat}}\left([\mathrm{Ca}^{2+}]\right) = \frac{J_{\text{ex}}^{\text{sat}}}{\Sigma_{\text{cone}}} \frac{[\mathrm{Ca}^{2+}]}{K_{\text{ex}} + [\mathrm{Ca}^{2+}]} \quad \text{and} \quad \mathrm{J}_{\text{cG}}^{\text{max}}([\mathrm{cG}]) = \frac{J_{\text{cGMP}}^{\text{max}}}{\Sigma_{\text{cone}}} \frac{[\mathrm{cG}]^{m_{cG}}}{K_{\text{cGMP}}^{m_{cG}} + [\mathrm{cG}]^{m_{cG}}} \tag{7}$$

The current values $J_{\text{ex}}^{\text{sat}}$, $J_{\text{cGMP}}^{\text{max}}$ are the maximum currents measured across the whole COS, respectively for either the exchanger as $[Ca^{2+}]$ becomes saturating or the cGMP-gated current as [cG] becomes saturating. Σ_{cone} is the surface area of the cone at the sliver. This normalization assumes that the channels are distributed uniformly on the sliver.

In (5) and (10), the striped bass measurement for J_{cG}^{max} is reported. A range for $J_{\text{ex}}^{\text{sat}}$ is also reported in (5). The upper value of this range is used in simulation.

2.0.11 Hydrolytic Efficiency of Activated PDE Dimer

In (11) Table 1, k_{cat}/K_M was reported to be $(5 * 10^3 \text{molecules}/s/(10\mu M))$. This value is within the range reported in (2) for mouse rod.

2.0.12 Surface Hydrolysis Rates of cGMP

The dark surface hydrolysis rate of cGMP, $k_{\sigma,\text{hyd}}$, may be computed from Eq. 23 in (2):

$$\begin{aligned} k_{\sigma,\text{hyd}} = \frac{\eta\beta_{\text{dark}}}{[PDE]_\sigma} &= \frac{(.0075\mu m)\,(67s^{-1})}{1000 \text{ PDE molecules per } \mu m^2} \\ &= 5.02 * 10^{-4} \mu m^3 s^{-1} \end{aligned}$$

The light activated surface hydrolysis rate of cGMP, $k_{\sigma,\text{hyd}}^*$, may be computed from the expression just after Eq. 24 in (2):

$$k_{\sigma,\text{hyd}}^* = \frac{k_{\text{cat}}/K_M}{N_{\text{Av}} B_{\text{cGMP}}} = \frac{\left(500\mu M^{-1} s^{-1}\right)}{6.02 * 10^{23}/mol} = .83\mu m^3 s^{-1}$$

2.0.13 Decay of Rhodopsin

Rhodopsin activity is mediated through phosphorylation by rhodopsin kinase and arrestin binding. In (12), a continuous time markov chain (CTMC) framework is developed to account for the stochasticity of rhodopsin shutoff in rods. In the case of carp cones, it has been found that total kinase activity is much higher than in rods (13). In principle same CTMC framework may be used for both rods and cones while the parameters decribing cone opsin shut-off differ. The rate at which an opsin with $i-1$ phosphorylations acquires an ith is

$$\lambda_i = (n_{\text{step}} - i)\lambda_0 \text{ for } i = 1, \ldots n_{\text{step}}.$$

The λ_0 value was estimated by scaling the mouse rod value in (12) by the ratio of carp cone GRK to carp rod GRK found in (14). The parameter n_{step} counts the number of phosphorylations an opsin can undergo beginning at the first step with 0 phosphorylations. Arrestin binds an opsin in the ith phosphorylation state with rate μ_i.

For present simulations a single step to arrestin shutoff was chosen and μ_0 was taken as the mouse rod decay rate k_R in (2). This was done due to a shortage of experimental measurements of arrestin binding phosphorylated cone opsin. For these choices the CTMC framework approximates an exponential decay model, which is the reasoning for substituting k_R for μ_0.

2.0.14 Catalytic Activity of Phosphorylated Opsin

Following (12) the activation rate of G-protein from opsin R^* is assumed to decrease exponentially with incremental phosphorylation. This is described by the relation

$$\nu_i = \nu_{RG} e^{-k_v(i-1)} \text{ for } i = 1, \dots, n_{\text{step}}.$$

The value k_v for rods reported in (12) was used in simulations. The rate ν_{RG} was taken from (11).

2.0.15 Michaelis-Menten and Hill Constants

K_{cyc} is the Michaelis-Menten constant for cyclase appearing in equation (6), and the value reported in (5) is used. The value of m_{cGMP} is also taken from (5). For K_{cG} and m_{cyc}, the mouse rod values reported in (2) were taken.

K_{ex} is used as an adjusted quantity for fit in Table 3 of (5). There a value of 19nm was obtained. In our simulations K_{ex}'s value of $0.69\mu M$ was chosen to fit the dark current of numerical simulations to the value $14.95pA \sim 15pA$. This K_{ex} value is much closer to the mouse rod range of $0.9 - 1.6\mu M$ reported in (2) than the adjusted-to-fit value value reported in (5).

2.0.16 Geometric Constants

From the measurements of ϵ and $\nu_\epsilon = \nu * \nu_\epsilon$, it follows that ν is taken as unity. The number of chambers may be computed from H, ν and ϵ through the relation

$$n = \frac{H}{\nu\epsilon + \epsilon} = 15\mu m/(2 * .015\mu m) = 500$$

2.0.17 Effector Surface Density

The highest reported mouse rod value of (2) was taken in simulations.

3 Simulated Effects of Reduced PDE Inhibition

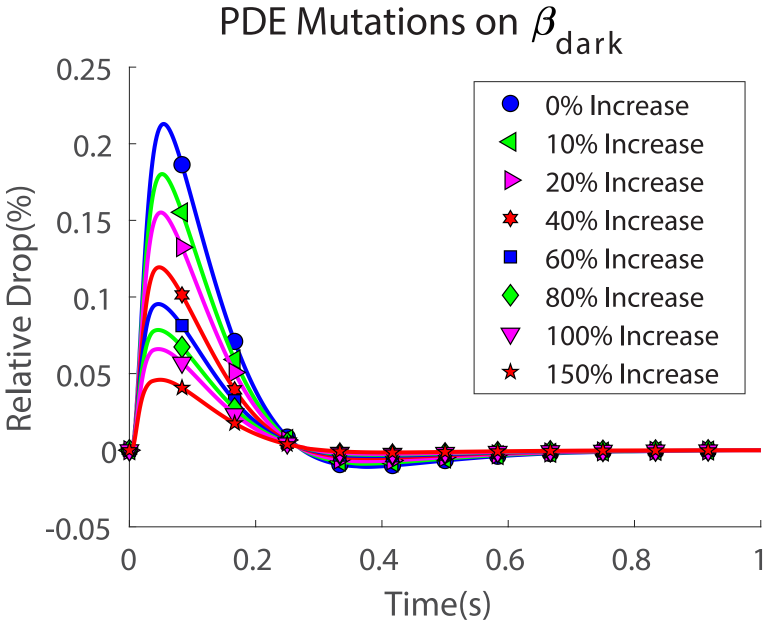

Figure 1: Simulated effect on cone's ten photon response of decreasing PDE inhibition by increasing dark hydrolysis parameter β_{dark}. Certain retinal rod disorders are known to decrease PDE inhibition (15–17).

To simulate the effects of reduced PDE inhibition, as in certain retinal rod disorders (15–17), the dark hydrolysis parameter $\beta_{\rm dark}$ has been relatively increased and the effect on the cone's ten photon response shown. The numerical findings support the conclusion that disorders which increase the basal activity of PDE diminish the photoreceptor's sensitivity to light.

4 Biochemistry Drives Differences Between Rods and Cones in their HOM vs N-HOM's Drop-Relative-Errors

One observes that the relative errors in drop for the cone homogenized model are bigger than the relative errors in drop for the rod homogenized model. A back of the envelope calculation can explain this empirical observation.

The absolute error in drop between the homogenized and nonhomogenized models is given by the difference of the peak drops between HOM, drop_h, and N-HOM, drop_{nh}: $e_a = |\text{drop}_h - \text{drop}_{nh}|$. The relative error is given by $e_r = 100\,|\text{drop}_{\rm h} - \text{drop}_{\rm nh}|\,/\text{drop}_{\rm nh}$. From this

$$\frac{e_{\rm r,cone}}{e_{\rm r,rod}} = \frac{100[e_{\rm a,cone}/\text{drop}_{\rm nh,cone}]}{100[e_{\rm a,rod}/\text{drop}_{\rm nh,rod}]} = \left(\frac{\text{drop}_{\rm nh,rod}}{\text{drop}_{\rm nh,cone}}\right)\left(\frac{e_{\rm a,cone}}{e_{\rm a,rod}}\right)$$

Differences in rod and cone biochemistry ensure that the rod drop is much larger than the cone drop, and so, for example, even if the homogenized and nonhomogenized model attain the same absolute error on both rods and cones, still the cone relative error will be substantially larger than the rod relative error.

Verifying Text Eq (2)

The following Maple worksheet is to verify the proportion of vol $(\bigcup C_j)$ in the cone. One minus this number is the value reported in Text Eq (2):

Maple Output

```
> with('LinearAlgebra'):
```

Chamber lengths of interdiscal and discal spaces along z-axis:

$[I_0 = 1/2\nu\epsilon \; C_1 = \epsilon]$ ... $[I_{j-1} = \nu\epsilon \; C_j = \epsilon]$ ... $[I_{n-1} = \nu\epsilon \; C_n = \epsilon]$ $[I_n = 1/2\nu\epsilon]$

For any cone chamber of small and large radius r, R and height H, we compute its volume. Its edge may be taken through (r,0) and (R,H):

y(x) = H/(R-r)*(x-r)

As a region of revolution, its volume is given by formula

$$V = \int_0^H \pi x(y)^2 dy$$

V_ch := int(pi*((R-r)/H*y + r)^2,y=0..H);

$$V\_ch := 1/3\,\pi\,(R-r)^2\,H + \pi\,r\,(R-r)\,H + \pi\,r^2 H$$

The lowest height of the chamber C_j is $1/2\nu\epsilon + (j-1)(\nu\epsilon + \epsilon)$

The upper height of the chamber C_j is $1/2\nu\epsilon + (j-1)(\nu\epsilon + \epsilon) + \epsilon$.

The corresponding x-values to those heights will be the small and large radius values for the chambers.

```
> rlower := j -> (R-r)/H*(
> 1/2*nu*epsilon+(j-1)*(nu*epsilon + epsilon)
> ) + r:
> rupper := j -> (R-r)/H*(
> 1/2*nu*epsilon+(j-1)*(nu*epsilon + epsilon) + epsilon
> ) + r:
```

Next compute vol$(\bigcup C_j)$.

```
> V_C :=sum(
> 1/3*pi*epsilon*(rupper(j) - rlower(j))^2 +
> pi*rlower(j)*(rupper(j)-rlower(j))*epsilon +
> pi*epsilon*(rlower(j))^2,
> j=1..n
> ):
> Nearultimate:=simplify(V_C/V_ch):
```

Apply the identity $n(\nu\epsilon + \epsilon) = H$, ie $n\epsilon = H/(1+\nu)$

```
> Penultimate:=collect(subs(n = H/(epsilon*(1+nu)),Nearultimate),epsilon);
```

$$\text{Penultimate} := \frac{\left(-1/4\,\nu^2 - \nu/2\right)(R-r)^2\,\epsilon^2}{H^2\,(\nu+1)\,(R^2+Rr+r^2)} + 3\,\frac{1}{H^2\,(\nu+1)\,(R^2+Rr+r^2)} * \cdots$$

$$\left(1/3\,\left(\frac{H^2\nu^2}{(\nu+1)^2} + 2\,\frac{H^2\nu}{(\nu+1)^2} + \frac{H^2}{(\nu+1)^2}\right)(R-r)^2 + H^2 r\,(R-r) + H^2 r^2\right)$$

The nonleading term is seen to be $O(\epsilon^2)$. Now simplify the leading term.

```
> simplify(coeff(Penultimate,epsilon,0));
```

$$(\nu+1)^{-1}$$

References

[1] Takemoto, N., S. Tachibanaki, and S. Kawamura, 2009. High cGMP synthetic activity in carp cones. *Proc. Natl. Acad. Sci. U.S.A.* 106:11788–11793.

[2] Shen, L., G. Caruso, P. Bisegna, D. Andreucci, V. V. Gurevich, H. E. Hamm, and E. DiBenedetto, 2010. Dynamics of mouse rod phototransduction and its sensitivity to variation of key parameters. *IET Sys. Biol.* 4:12–32.

[3] Koshitani, Y., S. Tachibanaki, and S. Kawamura, 2014. Quantitative aspects of cGMP phosphodiesterase activation in carp rods and cones. *J. Biol. Chem.* 289:2651–2657.

[4] Korenbrot, J. I., 2012. Speed, sensitivity, and stability of the light response in rod and cone photoreceptors: facts and models. *Prog Retin Eye Res* 31:442–466.

[5] Korenbrot, J. I., 2012. Speed, adaptation, and stability of the response to light in cone photoreceptors: the functional role of Ca-dependent modulation of ligand sensitivity in cGMP-gated ion channels. *J. Gen. Physiol.* 139:31–56.

[6] Arinobu, D., S. Tachibanaki, and S. Kawamura, 2010. Larger inhibition of visual pigment kinase in cones than in rods. *J. Neurochem.* 115:259–268.

[7] Sampath, A. P., H. R. Matthews, M. C. Cornwall, J. Bandarchi, and G. L. Fain, 1999. Light-dependent changes in outer segment free-Ca2+ concentration in salamander cone photoreceptors. *J. Gen. Physiol.* 113:267–277.

[8] Ohyama, T., A. Picones, and J. I. Korenbrot, 2002. Voltage-dependence of ion permeation in cyclic GMP-gated ion channels is optimized for cell function in rod and cone photoreceptors. *J. Gen. Physiol.* 119:341–354.

[9] Pugh, E. N. J., and T. D. Lamb, 2000. Phototransduction in vertebrate rods and cones: molecular mechanisms of amplification, recovery and light adaptation, Elsevier Science, St. Louis, volume 3 of *Handbook of Biological Physics*, chapter 5, 183–255.

[10] Rebrik, T. I., E. A. Kotelnikova, and J. I. Korenbrot, 2000. Time course and Ca(2+) dependence of sensitivity modulation in cyclic GMP-gated currents of intact cone photoreceptors. *J. Gen. Physiol.* 116:521–534.

[11] Holcman, D., and J. I. Korenbrot, 2005. The limit of photoreceptor sensitivity: molecular mechanisms of dark noise in retinal cones. *J. Gen. Physiol.* 125:641–660.

[12] Caruso, G., P. Bisegna, L. Lenoci, D. Andreucci, V. V. Gurevich, H. E. Hamm, and E. DiBenedetto, 2010. Kinetics of rhodopsin deactivation and its role in regulating recovery and reproducibility of rod photoresponse. *PLoS Comput. Biol.* 6:e1001031.

[13] Tachibanaki, S., Y. Shimauchi-Matsukawa, D. Arinobu, and S. Kawamura, 2007. Molecular mechanisms characterizing cone photoresponses. *Photochem. Photobiol.* 83:19–26.

[14] Kawamura, S., and S. Tachibanaki, 2012. Explaining the functional differences of rods versus cones. *Wiley Interdisciplinary Reviews: Membrane Transport and Signaling* 1:675–683. `https://onlinelibrary.wiley.com/doi/abs/10.1002/wmts.8`.

[15] Tsang, S. H., M. L. Woodruff, C. S. Lin, B. D. Jacobson, M. C. Naumann, C. W. Hsu, R. J. Davis, M. C. Cilluffo, J. Chen, and G. L. Fain, 2012. Effect of the ILE86TER mutation in the Î3 subunit of cGMP phosphodiesterase (PDE6) on rod photoreceptor signaling. *Cell. Signal.* 24:181–188.

[16] Muradov, K. G., A. E. Granovsky, and N. O. Artemyev, 2003. Mutation in rod PDE6 linked to congenital stationary night blindness impairs the enzyme inhibition by its gamma-subunit. *Biochemistry* 42:3305–3310.

[17] Gal, A., U. Orth, W. Baehr, E. Schwinger, and T. Rosenberg, 1994. Heterozygous missense mutation in the rod cGMP phosphodiesterase beta-subunit gene in autosomal dominant stationary night blindness. *Nat. Genet.* 7:64–68.
